# Identification and Comparative Analysis of H_2_O_2_-Scavenging Enzymes (Ascorbate Peroxidase and Glutathione Peroxidase) in Selected Plants Employing Bioinformatics Approaches

**DOI:** 10.3389/fpls.2016.00301

**Published:** 2016-03-22

**Authors:** Ibrahim I. Ozyigit, Ertugrul Filiz, Recep Vatansever, Kuaybe Y. Kurtoglu, Ibrahim Koc, Münir X. Öztürk, Naser A. Anjum

**Affiliations:** ^1^Department of Biology, Faculty of Science and Arts, Marmara UniversityIstanbul, Turkey; ^2^Department of Crop and Animal Production, Cilimli Vocational School, Düzce UniversityDüzce, Turkey; ^3^Department of Molecular Biology and Genetics, Faculty of Science, Istanbul Medeniyet UniversityIstanbul, Turkey; ^4^Department of Molecular Biology and Genetics, Faculty of Science, Gebze Technical UniversityKocaeli, Turkey; ^5^Botany Department/Center for Environmental Studies, Ege UniversityIzmir, Turkey; ^6^Faculty of Forestry, Universiti Putra MalaysiaSelangor, Malaysia; ^7^Centre for Environmental and Marine Studies and Department of Chemistry, University of AveiroAveiro, Portugal

**Keywords:** ROS, signal transduction, antioxidant, peroxisome, chloroplast, mitochondria

## Abstract

Among major reactive oxygen species (ROS), hydrogen peroxide (H_2_O_2_) exhibits dual roles in plant metabolism. Low levels of H_2_O_2_ modulate many biological/physiological processes in plants; whereas, its high level can cause damage to cell structures, having severe consequences. Thus, steady-state level of cellular H_2_O_2_ must be tightly regulated. Glutathione peroxidases (GPX) and ascorbate peroxidase (APX) are two major ROS-scavenging enzymes which catalyze the reduction of H_2_O_2_ in order to prevent potential H_2_O_2_-derived cellular damage. Employing bioinformatics approaches, this study presents a comparative evaluation of both GPX and APX in 18 different plant species, and provides valuable insights into the nature and complex regulation of these enzymes. Herein, (a) potential GPX and APX genes/proteins from 18 different plant species were identified, (b) their exon/intron organization were analyzed, (c) detailed information about their physicochemical properties were provided, (d) conserved motif signatures of GPX and APX were identified, (e) their phylogenetic trees and 3D models were constructed, (f) protein-protein interaction networks were generated, and finally (g) GPX and APX gene expression profiles were analyzed. Study outcomes enlightened GPX and APX as major H_2_O_2_-scavenging enzymes at their structural and functional levels, which could be used in future studies in the current direction.

## Introduction

Reactive oxygen species (ROS), once perceived as toxic by-products, were known to cause oxidative damage in cells (Mittler et al., 2004; Suzuki and Mittler, 2006). Later, novel regulatory roles of these species were revealed in a wide range of biological processes such as cell signaling, growth, development, programmed cell death, and plant responses to various biotic/abiotic stress factors (Mullineaux and Karpinski, 2002; Uzilday et al., 2014). H_2_O_2_ is an endogenous ROS species known to play a dual role in plants, where it is beneficial at low concentrations but lethal at higher levels (Petrov and Van Breusegem, 2012). Nevertheless, at steady state levels, H_2_O_2_ acts as signaling molecule inducing the signal transduction mechanism to produce various cellular responses. Interestingly, pre-treatment of plants with H_2_O_2_ makes them more tolerant to biotic/abiotic stresses (Hossain et al., 2015). H_2_O_2_ was also noted for its regulatory functions in photosynthesis, cell cycle, development, senescence, and apoptosis (Mittler et al., 2004; Petrov and Van Breusegem, 2012). H_2_O_2_ has been accepted as a central component of signal transduction pathways in plant-adaptation to altered environmental conditions as it is both the only ROS with high permeability across membranes (that enables the transport of signals to distant sites) and its high stability when compared to other ROS with ~1 ms half-life (Bienert et al., 2007; Dynowski et al., 2008; Petrov and Van Breusegem, 2012). On the other hand, when the delicate balance between production and scavenging of H_2_O_2_ is disturbed, its overproduction results in significant damage to cell structures (Anjum et al., 2015; Sofo et al., 2015). To overcome H_2_O_2_-related cellular damage, aerobic organisms have developed various antioxidant machineries with enzymatic and non-enzymatic components. Ascorbate peroxidase (APX), glutathione peroxidase (GPX), and catalase (CAT) are the main enzymes responsible for suppressing toxic levels of H_2_O_2_ (Apel and Hirt, 2004). However, APX may have pivotal roles in ROS-scavenging because even very low concentrations are sufficient for H_2_O_2_ decomposition (Anjum et al., 2014; Sofo et al., 2015).

APX (EC, 1.11.1.11) belongs to the plant-type heme peroxidase superfamily in plants (Lazzarotto et al., 2011). Genome-wide studies demonstrated that APX in higher plants is encoded by multigenic families. *Arabidopsis* was reported to contain nine APX genes; whereas, rice has eight and tomato seven (Chew et al., 2003; Teixeira et al., 2004; Najami et al., 2008). Different isoforms are classified into sub-families according to their subcellular localization. Transmembrane domains in N- and C- terminal regions, as well as organelle-specific target molecules are the primary determinants in target localization of APXs (Ishikawa et al., 1998; Negi, 2011). Among nine *APX* genes identified in *Arabidopsis*, three were found to be encoded in cytosol whereas the other six were distributed in stroma, thylakoid, and peroxisome (Chew et al., 2003; Mittler et al., 2004). In rice, chloroplastic isoforms were expressed by three genes, cytosolic and peroxisomal forms were both encoded by two genes, and one gene was for the mitochondrial APX (Teixeira et al., 2006; Anjum et al., 2014). APX activity was also reported to increase under various stress conditions. For example, APX is differentially upregulated in response to heavy metal, drought, water, and heat stress (Sharma and Dubey, 2005; Koussevitzky et al., 2008; Yang et al., 2008; Anjum et al., 2014). In a previous study, Arg-38, Glu-65, Asn-71, and Asp208 residues were reported to be conserved among the entire APX family and known to be important in ligand (heme)-binding (Welinder, 1992). In addition to enzymatic properties, structural investigations on catalytic domains of the enzymes have been also performed. Three-dimensional structures of cAPX, sAPX, and their substrates showed the relationship between loop structure and stability in the absence of ascorbate (AsA; Yabuta et al., 2000; Anjum et al., 2014). The mitochondrial and chloroplastic APXs (< 30 s) have shorter half inactivation times (>1 h) compared to cytosolic and peroxisomal isoforms, which makes them more sensitive in either low concentrations or the absence of AsA (Caverzan et al., 2012; Anjum et al., 2014). Another important enzyme in H_2_O_2_-scavenging is the GPX from the non-heme containing peroxidase family (Bela et al., 2015). In *Arabidopsis*, eight *GPX* genes were reported (Milla et al., 2003; Koua et al., 2009). Based on *in silico* analysis, GPXs were predicted in chloroplast, mitochondria, cytosol, and ER localizations (Rouhier and Jacquot, 2005), and demonstrated high level of sequence similarity with strictly conserved cysteines and motifs (Dietz, 2011). Plant GPXs have cysteine residue in their active site (Koua et al., 2009), which is functional in both glutathione (GSH) and thiol peroxidase classes of the non-heme family. GPXs were also reported to be involved in stress responses. Many studies have demonstrated the significant increase in mRNA levels of GPXs under various abiotic/abiotic stress conditions such as oxidative stress, pathogen attack, metal, cold, drought, and salt (Navrot et al., 2006; Diao et al., 2014; Fu, 2014; Gao et al., 2014). For example, GPX genes were found to be upregulated under excess H_2_O_2_ and cold stresses in rice (Passaia et al., 2013). Transcriptome analysis indicated high level of GPX transcripts in dehydrated *Glycine max* samples (Criqui et al., 1992; Ferreira Neto et al., 2013). Several transgenic studies also supported the proposed function of GPXs. For example, the overexpression of GPX in its transgenic tomato resulted in higher tolerance against abiotic stress (Herbette et al., 2011). In addition to stress response, GPXs are also thought to regulate cellular redox homeostasis by modulating the thiol-disulfide balance (Bela et al., 2015). GPX expression was found to be highly upregulated to maintain redox homeostasis under oxidative stress which helped *Brassica rapa* to adapt long-term spaceflight (Sugimoto et al., 2014).

A scan of contemporary literature reveals a paucity of information on the identification and comparative analysis of GPX and APX in model and economically important food crops. Given the above, employing bioinformatics approaches, efforts were made in this study (a) to identify potential GPX and APX genes/proteins from 18 different plant species, (b) to analyze their exon/intron organization, (c) to provide detailed information about their physico-chemical properties, (d) to identify conserved motif signatures of GPX and APX, (e) to construct their phylogenetic trees and 3D models, (f) to generate protein-protein interaction networks, and finally (g) to analyze GPX and APX gene expression profiles.

## Materials and methods

### Retrieval of GPX and APX genes/proteins

Eight *Arabidopsis* GPX reference protein sequences such as GPX1 (P52032.2), GPX2 (O04922.1), GPX3 (O22850.1), GPX4 (Q8L910.1), GPX5 (Q9LYB4.1), GPX6 (O48646.2), GPX7 (Q9SZ54.2), and GPX8 (Q8LBU2.1), as well as and eight *Arabidopsis* APX reference sequences such as APX1 (Q05431.2), APX2 (Q1PER6.3), APX3 (Q42564.1), APX4 (P82281.2), APX5 (Q7XZP5.2), APX6 (Q8GY91.1), APXT (Q42593.2), and APXS (Q42592.2) were obtained from UniProtKB/Swiss-Prot database of NCBI (Romiti, 2006). These reference sequences were queried in proteome datasets of selected 18 plant species: *Arabidopsis thaliana* (L.) Heynh., *Brachypodium distachyon* (L.) P. Beauv., *Brasica rapa* L., *Chlamydomonas reinhardtii* P. A. Dang., *Cucumis sativus* L., *Eucalyptus grandis* W. Hill ex Maiden, *Glycine max* (L.) Merr., *Gossypium raimondii* Ulbr., *Medicago truncatula* Gaertn., *Oryza sativa* L., *Phaseolus vulgaris* L., *Physcomitrella patens* (Hedw.) Bruch & Schimp., *Populus trichocarpa* Torr. & A.Gray ex. Hook., *Prunus persica* (L.) Batsch, *Solanum lycopersicum* L., *Sorghum bicolor* (L.) Moench, *Vitis vinifera* L., and *Zea mays* L., all found in the Phytozome v.10.3 database (Goodstein et al., 2012). After sequences were obtained, the Hidden Markov Model (HMM) search of protein sequences were performed by Pfam (http://pfam.sanger.ac.uk) to confirm the protein domain families (Finn et al., 2016). Species were arbitrarily selected to represent the main plant groups such as monocots, dicots, and lower plants.

### Analysis of GPX and APX genes/proteins

Physicochemical properties of GPX and APX proteins were determined by using ProtParam tool (Gasteiger et al., 2005). Sub-cellular localization was predicted by CELLO (Yu et al., 2006) and WoLF PSORT (Horton et al., 2007) servers. Exon-intron organization of *GPX/APX* genes was analyzed by using a GSDS server (Hu et al., 2014). The Conserved motif structure of GPX/APX sequences was analyzed using the MEME tool with the following parameter settings: maximum number of motifs to find, 5; minimum width of motif, 6 and maximum width of motif, 50 (Bailey et al., 2009). Protein sequences were aligned by ClustalW (Thompson et al., 1994) and phylogenies were constructed by MEGA 6 (Tamura et al., 2013) with the maximum likelihood (ML) method for 1.000 bootstraps. The gene duplication events were detected using the following criteria: (a) length of alignable sequence covers >75% of the longer gene, and (b) similarity of aligned regions is >75% (Gu et al., 2002). The expression data of APX and GPX genes at anatomical and developmental levels were retrieved from the Genevestigator database (Hruz et al., 2008). 3D models of APX/GPXs were predicted by using the Phyre^2^ server (Kelley and Sternberg, 2009). Model validation was performed by Rampage Ramachandran plot analysis (Lovell et al., 2003). 3D structure comparisons were done by calculating RMSD values of models using the CLICK server employing α-carbon superposition (Nguyen et al., 2011). Putative interaction partners of APX/GPXs were predicted with the STRING server (Franceschini et al., 2013) and an interactome network was generated using cytoscape (Smoot et al., 2011).

## Results and discussion

H_2_O_2_ plays double roles in plants and modulates various crucial metabolic processes (Petrov and Van Breusegem, 2012). However, its increased levels can cause severe damage to cell structures; hence, steady-state level of cellular H_2_O_2_ is required to be tightly regulated (Anjum et al., 2014, 2015; Sofo et al., 2015). GPX and APX are two major ROS-scavenging enzymes which catalyze the reduction of H_2_O_2_ to prevent H_2_O_2_-derived cellular damage. In order to understand the structural, functional as well as evolutionary aspects of GPX and APX, employing bioinformatics approaches, this study attempted to present comparative analyses of putative GPX and APX homologs identified from18 plant species.

### Analysis of GPXs

#### Retrieval of GPX genes/proteins

Eight potential *Arabidopsis* GPX protein sequences, namely GPX1-8, obtained from the UniProtKB/Swiss-Prot database of NCBI were used as queries in Phytozome database to retrieve the very close homologs of GPX sequences in 18 plant species. In the selection of GPX homologs from blastp hits, very strict criteria (only the highest hit sequence) was applied to avoid the redundant sequences and alternative splices of the same gene. A total of 87 GPX sequences were identified from the protein datasets of 18 plant species. These include; 8 genes for *A. thaliana*, 4 genes for *B. distachyon*, 8 genes for *B. rapa*, 1 gene for *C. reinhardtii*, 6 genes for *C. sativus*, 5 genes for *E. grandis*, 5 genes for *G. max*, 6 genes for *G. raimondii*, 5 genes for *M. truncatula*, 5 genes for *O. sativa*, 5 genes for *P. vulgaris*, 2 genes for *P. patens*, 5 genes for *P. trichocarpa*, 5 genes for *P. persica*, 5 genes for *S. lycopersicum*, 4 genes for *S. bicolor*, 5 genes for *V. vinifera*, and 3 genes for *Z. mays* (Table 1). Then, genomic, transcript, CDS, and protein sequences of identified 87 GPX sequences were retrieved for further analyses.

**Table 1:** **List of H_**2**_O_**2**_-scavenging enzyme glutathione peroxidase (GPX) homologs from 18 plant species and their primary gene/protein features**.

**Species name**	**Phytozome gene ID**	**Gene/protein features of GPX sequences**							
		**Protein domain family^a^**	**Domain family description**	**Exon no.**	**Protein length**	**MW (KDa)**	***Theor. pI***	**Localization CELLO^b^**	**Localization WoLF PSORT^b^**
*Arabidopsis thaliana* (L.) Heynh.	AT1G63460	GSHPx (PF00255)	Glutathione peroxidase	6	167	19.0	5.11	Cyto	Cyto
	AT2G25080	GSHPx (PF00255)	Glutathione peroxidase	6	236	26.0	9.42	Chlo/Mito	Chlo
	AT2G31570	GSHPx (PF00255)	Glutathione peroxidase	6	169	18.9	5.60	Cyto	Cyto
	AT2G43350	GSHPx (PF00255)	Glutathione peroxidase	6	206	23.2	9.24	Mito/Plas	Chlo/Mito
	AT2G48150	GSHPx (PF00255)	Glutathione peroxidase	6	170	19.3	8.87	Cyto	Mito
	AT3G63080	GSHPx (PF00255)	Glutathione peroxidase	6	173	19.3	9.28	Extr/Chlo/Nucl	Chlo
	AT4G11600	GSHPx (PF00255)	Glutathione peroxidase	6	232	25.5	9.38	Mito/Chlo	Mito
	AT4G31870	GSHPx (PF00255)	Glutathione peroxidase	6	233	25.7	9.53	Chlo	Chlo
*Brachypodium distachyon* (L.) P.Beauv.	Bradi1g47140	GSHPx (PF00255)	Glutathione peroxidase	6	226	24.4	9.57	Chlo	Chlo
	Bradi1g61930	GSHPx (PF00255)	Glutathione peroxidase	6	198	22.4	7.56	Cyto	Cyto
	Bradi3g51010	GSHPx (PF00255)	Glutathione peroxidase	6	240	25.9	9.05	Chlo	Chlo
	Bradi5g18000	GSHPx (PF00255)	Glutathione peroxidase	6	168	18.4	6.31	Cyto/Chlo	Nucl/Chlo
*Brasica rapa* L.	Brara.B02692	GSHPx (PF00255)	Glutathione peroxidase	6	229	25.2	9.21	Mito	Chlo/Mito
	Brara.C02198	GSHPx (PF00255)	Glutathione peroxidase	6	197	21.9	8.55	Extr/Plas	Extr
	Brara.E00003	GSHPx (PF00255)	Glutathione peroxidase	6	170	19.2	9.05	Extr/Cyto	Chlo
	Brara.E01208	GSHPx (PF00255)	Glutathione peroxidase	6	169	18.9	6.34	Cyto	Cyto
	Brara.G01994	GSHPx (PF00255)	Glutathione peroxidase	6	176	19.5	9.15	Extr/Cyto/Nucl	Chlo
	Brara.I01234	GSHPx (PF00255)	Glutathione peroxidase	6	167	18.9	5.00	Cyto	Cyto
	Brara.I04448	GSHPx (PF00255)	Glutathione peroxidase	6	233	25.8	9.29	Mito/Chlo/Extr	Chlo
	Brara.K00392	GSHPx (PF00255)	Glutathione peroxidase	6	232	25.9	9.60	Mito/Extr	Chlo
*Chlamydomonas reinhardtii* P.A.Dang.	Cre03.g197750	GSHPx (PF00255)	Glutathione peroxidase	7	200	21.9	9.39	Mito	Chlo
*Cucumis sativus* L.	Cucsa.084960	GSHPx (PF00255)	Glutathione peroxidase	6	176	19.7	8.86	Cyto	Chlo
	Cucsa.094950	GSHPx (PF00255)	Glutathione peroxidase	6	204	23.4	8.55	Plas/Extr	Chlo
	Cucsa.184280	GSHPx (PF00255)	Glutathione peroxidase	6	170	19.0	8.66	Cyto/Extr	Cyto
	Cucsa.271420	GSHPx (PF00255)	Glutathione peroxidase	6	241	26.4	9.5	Chlo	Chlo
	Cucsa.303050	GSHPx (PF00255)	Glutathione peroxidase	6	241	26.8	9.28	Mito	Chlo/Mito
	Cucsa.303070	GSHPx (PF00255)	Glutathione peroxidase	6	170	19.2	5.21	Cyto	Cyto
*Eucalyptus grandis* W. Hill ex Maiden	Eucgr.A00257	GSHPx (PF00255)	Glutathione peroxidase	6	202	22.8	7.62	Extr/Chlo	Chlo/Vacu
	Eucgr.C02602	GSHPx (PF00255)	Glutathione peroxidase	6	247	26.9	9.53	Chlo	Chlo
	Eucgr.D01856	GSHPx (PF00255)	Glutathione peroxidase	6	170	19.4	5.16	Cyto	Cyto
	Eucgr.E00579	GSHPx (PF00255)	Glutathione peroxidase	6	250	27.3	9.16	Chlo	Chlo
	Eucgr.K03389	GSHPx (PF00255)	Glutathione peroxidase	6	170	18.9	9.02	Cyto	Chlo/Nucl
*Glycine max* (L.) Merr.	Glyma.03G151500	GSHPx (PF00255)	Glutathione peroxidase	6	170	19.0	9.45	Mito/Cyto	Chlo
	Glyma.05G240100	GSHPx (PF00255)	Glutathione peroxidase	6	199	22.7	7.54	Extr	Extr
	Glyma.08G013900	GSHPx (PF00255)	Glutathione peroxidase	6	167	18.9	5.09	Cyto	Chlo
	Glyma.11G024100	GSHPx (PF00255)	Glutathione peroxidase	6	167	18.5	5.88	Cyto	Cyto
	Glyma.17G223900	GSHPx (PF00255)	Glutathione peroxidase	6	234	26.3	9.40	Mito/Chlo	Chlo
*Gossypium raimondii* Ulbr.	Gorai.001G038600	GSHPx (PF00255)	Glutathione peroxidase	6	242	26.6	9.30	Chlo	Chlo
	Gorai.004G083200	GSHPx (PF00255)	Glutathione peroxidase	6	171	19.1	9.24	Nucl/Cyto/Extr	Nucl
	Gorai.004G087300	GSHPx (PF00255)	Glutathione peroxidase	6	208	23.6	5.51	Extr	Extr
	Gorai.004G211400	GSHPx (PF00255)	Glutathione peroxidase	6	166	18.4	6.73	Cyto	Chlo
	Gorai.006G186100	GSHPx (PF00255)	Glutathione peroxidase	6	168	18.7	6.73	Cyto	Cyto
	Gorai.008G246600	GSHPx (PF00255)	Glutathione peroxidase	6	168	19.1	4.59	Cyto	Chlo
*Zea mays* L.	GRMZM2G012479	GSHPx (PF00255)	Glutathione peroxidase	6	230	24.9	9.55	Mito	Chlo
	GRMZM2G144153	GSHPx (PF00255)	Glutathione peroxidase	6	168	18.4	6.58	Cyto	Chlo/Nucl
	GRMZM2G329144	GSHPx (PF00255)	Glutathione peroxidase	6	170	19.2	7.58	Cyto	Chlo
*Vitis vinifera* L.	GSVIVG01010737001	GSHPx (PF00255)	Glutathione peroxidase	6	167	18.6	5.53	Cyto	Cyto
	GSVIVG01011101001	GSHPx (PF00255)	Glutathione peroxidase	6	170	19.0	9.22	Cyto	Mito
	GSVIVG01019765001	GSHPx (PF00255)	Glutathione peroxidase	6	170	19.2	5.01	Cyto	Cyto
	GSVIVG01019766001	GSHPx (PF00255)	Glutathione peroxidase	6	168	18.6	6.73	Cyto	Chlo/Extr
	GSVIVG01035981001	GSHPx (PF00255)	Glutathione peroxidase	6	207	22.9	9.16	Chlo/Mito	Chlo
*Oryza sativa* L.	LOC_Os02g44500	GSHPx (PF00255)	Glutathione peroxidase	6	238	25.8	9.42	Chlo	Chlo
	LOC_Os03g24380	GSHPx (PF00255)	Glutathione peroxidase	6	169	19.2	8.80	Cyto	Cyto
	LOC_Os04g46960	GSHPx (PF00255)	Glutathione peroxidase	6	168	18.4	8.33	Cyto	Chlo
	LOC_Os06g08670	GSHPx (PF00255)	Glutathione peroxidase	6	234	25	9.51	Mito/Chlo	Chlo
	LOC_Os11g18170	GSHPx (PF00255)	Glutathione peroxidase	6	212	22.9	7.62	Chlo/Extr	Chlo
*Medicago truncatula* Gaertn.	Medtr1g014210	GSHPx (PF00255)	Glutathione peroxidase	6	236	26.4	9.32	Mito/Chlo	Chlo
	Medtr7g094600	GSHPx (PF00255)	Glutathione peroxidase	6	170	19.2	9.18	Cyto/Mito	Nucl
	Medtr8g098400	GSHPx (PF00255)	Glutathione peroxidase	6	172	19.3	4.82	Cyto	Chlo
	Medtr8g098410	GSHPx (PF00255)	Glutathione peroxidase	6	233	25.8	9.27	Mito	Chlo
	Medtr8g105630	GSHPx (PF00255)	Glutathione peroxidase	6	167	18.9	8.32	Plas	Chlo
*Physcomitrella patens* (Hedw.) Bruch & Schimp.	Phpat.004G103100	GSHPx (PF00255)	Glutathione peroxidase	6	247	26.7	9.24	Chlo/Extr	Chlo
	Phpat.017G045400	GSHPx (PF00255)	Glutathione peroxidase	1	170	19.1	8.30	Cyto	Cyto
*Phaseolus vulgaris* L.	Phvul.001G041100	GSHPx (PF00255)	Glutathione peroxidase	6	262	29.7	9.68	Mito	Chlo
	Phvul.001G149000	GSHPx (PF00255)	Glutathione peroxidase	6	168	18.8	9.31	Cyto/Nucl	Nucl
	Phvul.002G157200	GSHPx (PF00255)	Glutathione peroxidase	6	170	19.0	4.97	Cyto	Chlo/Nucl
	Phvul.002G288700	GSHPx (PF00255)	Glutathione peroxidase	6	230	25.6	8.76	Chlo/Mito	Chlo
	Phvul.002G322400	GSHPx (PF00255)	Glutathione peroxidase	6	198	22.5	5.94	Extr	Extr
*Populus trichocarpa* Torr. & A.Gray ex. Hook.	Potri.001G105100	GSHPx (PF00255)	Glutathione peroxidase	5	170	19.3	4.78	Cyto	Cyto
	Potri.003G126100	GSHPx (PF00255)	Glutathione peroxidase	6	238	26.2	9.29	Mito/Chlo	Chlo
	Potri.006G265400	GSHPx (PF00255)	Glutathione peroxidase	6	232	25.3	9.48	Chlo/Mito	Chlo
	Potri.007G126600	GSHPx (PF00255)	Glutathione peroxidase	6	203	22.8	6.83	Extr	Extr/Vacu
	Potri.014G138800	GSHPx (PF00255)	Glutathione peroxidase	6	170	18.9	9.15	Cyto/Extr	Chlo/Cyto
*Prunus persica (L.)* Batsch	ppa010584m.g	GSHPx (PF00255)	Glutathione peroxidase	6	244	26.7	9.33	Chlo	Chlo
	ppa010771m.g	GSHPx (PF00255)	Glutathione peroxidase	6	237	25.9	9.20	Mito	Chlo
	ppa011682m.g	GSHPx (PF00255)	Glutathione peroxidase	6	200	22.7	8.27	Extr/Cyto	Extr
	ppa012378m.g	GSHPx (PF00255)	Glutathione peroxidase	6	172	19.4	8.97	Cyto	Nucl/Cyto
	ppa012416m.g	GSHPx (PF00255)	Glutathione peroxidase	6	170	19.4	4.86	Cyto	Chlo
*Sorghum bicolor* (L.) Moench	Sobic.001G365800	GSHPx (PF00255)	Glutathione peroxidase	6	171	19.3	8.79	Cyto	Chlo
	Sobic.006G173900	GSHPx (PF00255)	Glutathione peroxidase	6	168	18.4	6.58	Cyto	Chlo/Nucl
	Sobic.010G067100	GSHPx (PF00255)	Glutathione peroxidase	6	232	24.9	9.50	Mito/Chlo	Chlo
	Sobic.K022000	GSHPx (PF00255)	Glutathione peroxidase	6	205	22.6	5.68	Cyto/Extr	Mito/Chlo
*Solanum lycopersicum* L.	Solyc06g073460.2	GSHPx (PF00255)	Glutathione peroxidase	6	167	18.9	6.37	Cyto	Chlo
	Solyc08g006720.2	GSHPx (PF00255)	Glutathione peroxidase	6	238	26.2	9.18	Chlo	Chlo
	Solyc08g080940.2	GSHPx (PF00255)	Glutathione peroxidase	6	239	26.7	9.16	Mito	Chlo
	Solyc09g064850.2	GSHPx (PF00255)	Glutathione peroxidase	6	170	19.0	9.33	Mito/Extr	Chlo
	Solyc12g056240.1	GSHPx (PF00255)	Glutathione peroxidase	6	170	19.3	4.97	Cyto	Cyto

a *Protein domain families were searched in Pfam database*.

b *Cyto, Cytosolic; Chlo, Chloroplastic; Mito, Mitochondrial; Vacu, Vacuolar; Nucl, Nuclear; Extr, Extracellular; Plas, Plasma membrane*.

*More than one localization specified in a single column also shows the significance of other entries in order*.

#### Sequence analysis of GPX genes/proteins

A total of 87 GPX homologs were identified in the protein datasets of 18 plant species using *Arabidopsis* GPX1-8 for homology search. Identified GPX homologs belonged to the GSHPx (PF00255) protein family. They encoded a polypeptide of 166–262 amino acids residues (average length 197.5) and 18.4–29.7 kDa molecular weight with 4.59–9.60 *pI* value. The sequence variations in analyzed GPXs primarily derived from the “transit peptide” residues between organelle and non-organelle related GPXs (Table 1). Studies of molecular cloning and sequencing in *A. thaliana* have reported that chloroplastic GPX1 and GPX7 consisted of 236 and 233 amino acids, respectively; the first 1–64 residues in GPX1 and 1–69 residues in GPX7 from N-terminal site contained the transit peptides (Mullineaux et al., 1998; Lin et al., 1999; Mayer et al., 1999). *Arabidopsis* GPX2 and GPX4 were reported to be 169 and 170 residues, respectively with cytosolic localization: thereby, they did not contain any transit peptide (Lin et al., 1999). *Arabidopsis* GPX3 and GPX6 were 206 and 232 residues, respectively, with mitochondrial localizations; the first 1–12 amino acids in GPX3 and 1–54 residues in GPX6 covered the transit peptide (Lin et al., 1999; Mayer et al., 1999). *Arabidopsis* GPX5 was 173 residues with probable ER or Plasma membrane localization, without transit peptide (Erfle et al., 2000). *Arabidopsis* GPX8 comprised of 167 amino acids with cytosolic or nuclear localization, without transit peptide (Theologis et al., 2000). In the present study, alignment analysis revealed that in chloroplastic/mitochondrial-related GPXs, the transit peptide sequences formed the first 50–90 amino acid residues from the N-terminal site while in extra cellular/plasma membrane-related GPXs, residues of the first 20–50 amino acid from N-terminal region contained the putative transit peptide. However, cytosolic sequences lacked of any putative transit residues (Supplementary Figure S1). Thus, analyzed GPX sequences were roughly categorized in three main groups based on their sequence length; the chloroplastic/mitochondrial related GPXs comprised the longer sequences (i), extra cellular/plasma membrane related GPXs formed the medium-sized sequences (ii), and cytosolic related GPXs included the shorter sequences (iii). In addition, the regions corresponding to the transit peptide sites in analyzed sequences did not demonstrate any particular patterns. The less-conserved transit peptide residues could be related with the functional diversities of GPXs at various targets. However, despite the variations in sequence length and transit peptide residues, transcripts of GPX homologs mainly contained the six exons. Therefore, it is reasonable to claim that analyzed GPX sequences could have highly-conserved protein sequences, preserved during the formation of various GPXs. The alignment analysis of 87 GPX protein homologs also confirmed this claim, demonstrating the presence of more conserved residues in the main sites of the active enzyme (Supplementary Figure S2). Moreover, to discern the conserved motif patterns in GPX sequences, the most conserved five motif sequences were searched in sets of 87 GPX homologs using MEME tool (Table 2). Motif 1 and 3 were the 50 amino acid residues, while the motif 2 was 41, motif 4 was 15, and motif 5 was 6 residues in length. Motif 1 and 3 were related with the GSHPx (PF00255) protein family and present in almost all GPX homologs. The presence of long conserved residues and their relation with the GSHPx family could indicate the highly conserved structures of GPX sequences at these sites between/among species.

**Table 2:** **Most conserved five motifs of glutathione peroxidase (GPX) homologs in 18 plant species**.

**Motif**	**Width**	**Identified site no**.	**Sequence**	**Protein domain family^a^**
1	50	87 of 87	KYKDQGFEILAFPCNQFGGQEPGTNEEIQQFACTRFKAEYPIFDKVDVNG	GSHPx (PF00255)
2	41	87 of 87	FGDRIKWNFTKFLVDKEGHVVDRYAPTTSPLQIEKDIQKLL	Not found
3	50	86 of 87	KSIHDFTVKDIRGNDVDLSIYKGKVLLIVNVASQCGMTNSNYTELNHLYE	GSHPx (PF00255)
4	15	87 of 87	NAAPLYKFLKSSKWG	Not found
5	6	63 of 87	MAASHS	Not found

a *Protein domain families have been searched in Pfam database*.

Furthermore, alignment analysis also demonstrated that Asn (N), Gly (G), Arg (R), Pro (P), Thr (T), Tyr (Y), Lys (K), Ala-Ser (AS), Cys-Gly (CG), Phe-Pro (FP), Glu-Pro (EP), Leu-Lys (LK), Lys-Phe (KF), Asn-Gly (NG), Asn-Gln-Phe (NQF), and Trp-Asn-Phe (WNF) residues were strictly conserved in all aligned sequences, indicating their potential functions in enzyme activity and/or stability (Supplementary Figure S3). To infer a functional relationship between these conserved residues and GPX sequences, we searched for the known catalytic residues of model organism *Arabidopsis* GPXs in the UniProtKB/Swiss-Prot database of NCBI (www.ncbi.nlm.nih.gov/protein). The following residues were designated in the database as potential catalytic residues for *Arabidopsis* GPX1-8: GPX1 (Cys-111, Gln-146, Trp-200), GPX2 (Cys-41, Gln-76, Trp-130), GPX3 (Cys-80, Gln-115, Trp-169), GPX4 (Cys-44, Gln-79, Trp-133), GPX5 (Cys-46, Gln-81, Trp-135), GPX6 (Cys-105, Gln-140, Trp-194), GPX7 (Cys-108, Gln-143, Trp-197), and GPX8 (Cys-41, Glu-76, Trp-130). Interestingly, residues that correspond to these catalytic sites in other analyzed sequences were found to be strictly conserved (Supplementary Figure S3). This shows that active sites of the enzyme are considerably conserved between species.

#### Phylogenetic analysis of GPXs

**Figure 1:**
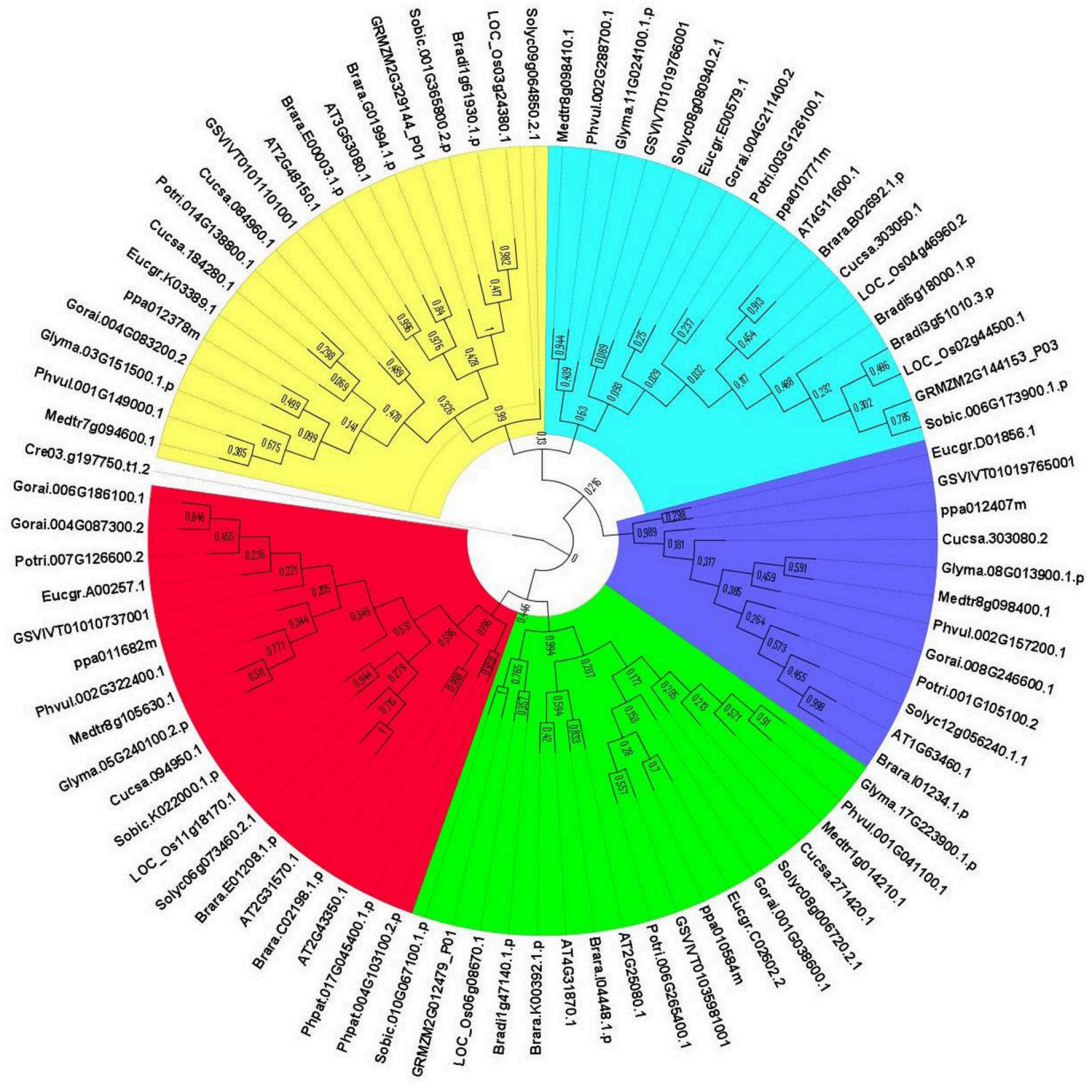
**Phylogenetic tree of glutathione peroxidase (GPX) homologs from 18 plant species**. Tree was constructed by MEGA 6 using Maximum likelihood (ML) method with 1000 bootstraps. Segment classification based on the consensus of two subcellular localization servers, CELLO and WoLF PSORT as well as tree topology for ambiguous sequences. Red segment includes cytosolic, extra cellular, and plasma membrane related GPXs, green segment contains mitochondrial and chloroplast related GPXs, blue segment only have cytosolic GPXs, cyan segment includes cytosolic, and chloroplast/mitochondrial related GPXs, yellow segment contains cytosolic/nuclear related GPXs, and non-colored segment has lower plant Chlamydomonas GPX with chloroplastic/mitochondrial relation.

The evolutionary relationships between identified GPX sequences were analyzed by MEGA 6 using the Maximum Likelihood (ML) method with 1000 bootstraps. The constructed phylogeny included all 87 GPX homologs to discover the phylogenetic distribution of sequences (Figure 1). The tree was divided into six major groups based on the tree topology, and each group was indicated with a different color segment. The red segment included cytosolic, extra cellular, and plasma membrane related GPXs, the green segment contained mitochondrial and chloroplast related GPXs, the blue segment only had cytosolic GPXs, the cyan segment included cytosolic and chloroplast/mitochondrial related GPXs, the yellow segment contained cytosolic/nuclear related GPXs, and the non-colored segment had lower plant *Chlamydomonas* GPX with a chloroplastic/mitochondrial relation. Annotation of each segment based on the consensus of two subcellular localization servers, CELLO and WoLF PSORT, as well as tree topology for ambiguous sequences. Mainly cytosolic, nuclear, extra cellular and plasma membrane related GPXs were clustered together, while chloroplast/mitochondrial related GPXs also cluster together. Therefore, the presence or absence of transit peptide residues was the main contributing entity in the phylogenetic distribution of GPX sequences. In addition, the presence of sequences with different subcellular localizations in the same group inferred the possibility of gene duplication events in the formation of various GPX sequences. Duplications in plant genomes could be either as small-scale such as tandem and segmental duplications, or as large-scale such as whole-genome duplications (Ramsey and Schemske, 1998). The segmental duplications are observed in different chromosomes whereas tandem duplications occur in the same chromosome (Liu et al., 2011). Several segmental duplications were identified between GPX pairs (Table 3). The presence of segmental duplications, particularly between sequences with various subcellular localizations may partly explain the possibility of gene duplication events in GPX formations.

**Table 3:** **The segmental duplication events in some glutathione peroxidase (GPX) pairs**.

**Species name**	**Segmental duplication pairs**
*Arabidopsis thaliana* (L.) Heynh.	AT2G25080-AT4G31870
	AT2G48150-AT3G63080
*Brachypodium distachyon* (L.) P.Beauv.	Bradi5g18000-Bradi3g51010
*Brasica rapa* L.	Brara.E00003-Brara.G01994
	Brara.I04448-Brara.K00392
*Gossypium raimondii* Ulbr.	Gorai.004G087300-Gorai.006G186100
	Gorai.004G211400-Gorai.008G246600
*Vitis vinifera* L.	GSVIVG01019765001-GSVIVG01019766001
*Oryza sativa* L.	LOC_Os04g46960-LOC_Os02g44500
*Physcomitrella patens* (Hedw.) Bruch & Schimp.	Phpat.017G045400-Phpat.004G103100
*Prunus persica* (L.) Batsch	ppa012416m.g-ppa010771m.g

#### Expression profile analysis of GPXs

**Figure 2:**
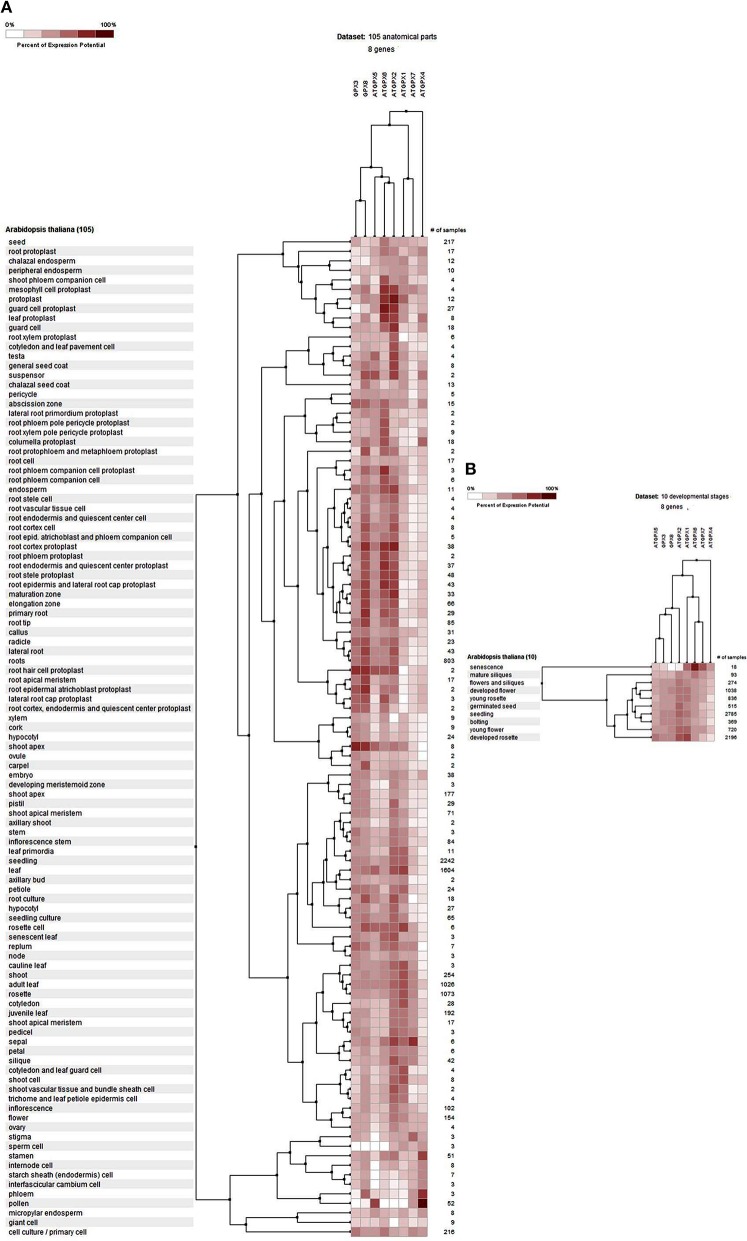
**Expression profile of ***Arabidopsis*** glutathione peroxidase ***GPX1-8*** genes at 105 anatomical parts (A) and 10 developmental stage levels (B)**. Genes and conditions with similar profiles were comparatively analyzed using hierarchical clustering tool with Euclidean distance method at Genevestigator platform.

The potential expression profile of *GPX* genes was analyzed at 105 anatomical parts and 10 developmental stage levels using model organism *A. thaliana* GPXs from Genevestigator platform (Figure 2). Eight *Arabidopsis* genes, namely GPX1 (AT2G25080), GPX2 (AT2G31570), GPX3 (AT2G43350), GPX4 (AT2G48150), GPX5 (AT3G63080), GPX6 (AT4G11600), GPX7 (AT4G31870), and GPX8 (AT1G63460) were retrieved from the “Affymetrix Arabidopsis ATH1 Genome Array” platform using the Genevestigator interface, and conditions and genes with similar profiles were comparatively analyzed using the Hierarchical clustering tool with the Euclidean distance method. At an anatomical part level (Figure 2A), analyzed *GPX1-8* genes were expressed in almost all 105 anatomical tissues in *Arabidopsis* plants. However, various root and leaf protoplast cells, seed-related tissues, and active growth zones demonstrated significantly higher GPX activity. This indicates that stress factors and/or active metabolism could lead to the up-regulation of various *GPX* genes in different tissues. Many studies have also showed that balance between production and scavenging of H_2_O_2_ could be disturbed by various biotic/abiotic stress factors or perturbations such as drought, salinity, pathogen attack, oxidative state of the cells (Apel and Hirt, 2004; Anjum et al., 2014, 2015; Sofo et al., 2015). Besides, a number of studies were also available demonstrating the functional roles of GPXs in plant stress resistance/tolerance. For example, a *GPX* gene in *Pennisetum glaucum* enhanced the drought and salinity stress tolerance (Islam et al., 2015). Citrus *GPX3* was reported to be essential in detoxification of ROS-induced cellular stresses as well as in *Alternaria alternata* pathogenesis (Yang et al., 2016). Silencing of mitochondrial *GPX1* in *O. sativa* demonstrated the impaired photosynthesis in response to salinity (Lima-Melo et al., 2016). Glutathione transferases and peroxidases were reported as key components in *Arabidopsis* salt stress-acclimation (Horváth et al., 2015). *GPX1* and *GPX3* in legume root nodules played a protective function against salt stress, oxidative stress, and membrane damage (Matamoros et al., 2015). Therefore, GPXs, which are the antioxidant enzymatic components of the cells, are consequently induced to suppress/eliminate the toxic levels of H_2_O_2_. The increased GPX activities in analyzed *Arabidopsis* tissues could thereby be derived from the increased H_2_O_2_ or H_2_O_2_-related products. In addition, clustering analysis of *GPX* genes showed that *GPX2, 3, 5, 6*, and *8* demonstrate relatively similar expression profiles compared to those of *GPX1, 4*, and *7*. At the developmental level (Figure 2B), the expression profiles of *Arabidopsis GPX1-8* genes were analyzed at 10 developmental stages, including senescence, mature siliques, flowers and siliques, developed flower, young rosette, germinated seed, seedling, bolting, young flower, and developed rosette. *GPX1-8* were relatively expressed in all developmental stages. However, the expression of *GPX*s in the senescence stage demonstrated slightly different patterns, particularly the mitochondrial *GPX6* gene had the highest expression profile compared to other developmental stages. This may have been caused by senescence-related cellular deteriorations, leading to the substantial metabolic or physiological changes that significantly affect the overall metabolic energy consumption. Therefore, it seems that the expression profiles of *GPX*s are highly associated with the metabolic state of the cells.

#### 3D structure analysis of GPXs

**Figure 3:**
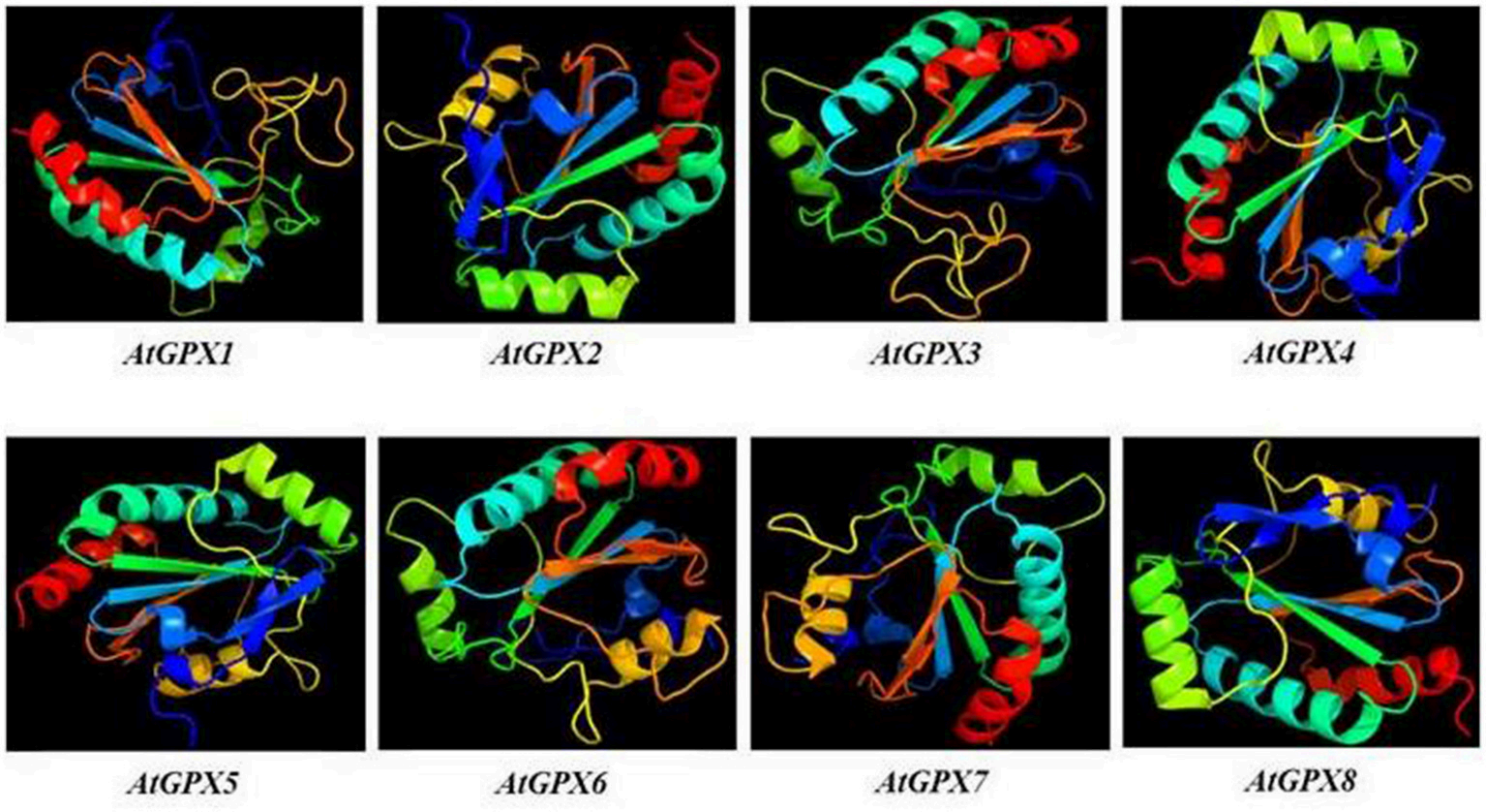
**3D models of predicted ***Arabidopsis*** glutathione peroxidase GPX1-8 sequences**. Models were constructed by using Phyre^2^ server for AT2G25080.1 (GPX1), AT2G31570.1 (GPX2), AT2G43350.1 (GPX3), AT2G48150.1 (GPX4), AT3G63080.1 (GPX5), AT4G11600.1 (GPX6), AT4G31870.1 (GPX7), and AT1G63460.1 (GPX8) sequences, and colored by rainbow from N- to C-terminus.

3D models of putative GPXs were constructed by using Phyre^2^ server for eight identified *Arabidopsis GPX1-8* gene sequences (Figure 3). These sequences were: AT2G25080.1 (*GPX1*), AT2G31570.1 (*GPX2*), AT2G43350.1 (*GPX3*), AT2G48150.1 (*GPX4*), AT3G63080.1 (*GPX5*), AT4G11600.1 (*GPX6*), AT4G31870.1 (*GPX7*), and AT1G63460.1 (*GPX8*). In modeling, three templates such as 2F8A:A (*GPX1, GPX3, GPX6*, and *GPX7*), 2V1M:A (*GPX2* and *GPX5*), and 2P5Q:A (*GPX4* and *GPX8*) were used to maximize the alignment coverage, percentage identity and confidence for submitted sequences. Predicted models demonstrated the ≥98% of residues in allowed region in Ramachandran plot analysis, indicating that constructed models were fairly in good quality. To find out the structural divergence/similarity in generated models, the structures were superposed to calculate the percentage of structural overlap and RMSD values (Table 4). GPX1-GPX3, GPX4-GPX8, and GPX6-GPX7 pairs demonstrated the highly conserved structural overlap (100%) with 0.14, 0.00, and 0.03 RMSD values, respectively. The each designated pair also belonged to either chloroplastic/mitochondrial or cytosolic form, indicating their functional similarities with minor specifications. In addition, GPX1-GPX6 and 7, GPX2-GPX5, and GPX3-GPX6 and 7 pairs showed very high structural similarity with ≥94 structural overlaps. Despite the highly conserved structures of *Arabidopsis* GPX members, some minor variations were also present. It seems that these divergences in GPXs may not change the protein-3D structure, however, they could attribute the new functional roles to catalytic activities.

**Table 4:** **Structural overlap (%)/RMSD values in superposed ***Arabidopsis*** glutathione peroxidases (GPXs)**.

	**GPX1**	**GPX2**	**GPX3**	**GPX4**	**GPX5**	**GPX6**	**GPX7**	**GPX8**
GPX1	–	88.68/1.03	100.00/0.14	91.19/1.10	89.94/0.91	94.34/0.24	94.34/0.24	91.19/1.10
GPX2	88.05/0.89	–	88.68/0.93	93.75/0.66	99.38/0.15	91.82/0.92	91.82/0.92	94.38/0.71
GPX3	100.00/0.14	89.94/1.10	–	90.57/0.90	90.57/0.95	94.34/0.28	94.34/0.28	90.57/0.90
GPX4	89.94/1.07	93.75/0.67	91.19/0.96	–	95.65/0.67	94.97/0.97	94.97/0.97	100.00/0.00
GPX5	90.57/0.89	99.38/0.15	90.57/0.91	95.65/0.66	–	94.34/0.91	94.34/0.91	95.65/0.67
GPX6	94.34/0.24	92.45/1.04	94.34/0.28	95.60/1.07	94.97/1.04	–	100.00/0.03	95.60/1.06
GPX7	94.34/0.24	92.45/1.05	94.34/0.28	95.60/1.06	94.97/1.04	100.00/0.03	–	95.60/1.06
GPX8	91.19/0.99	95.00/0.75	91.19/0.98	100.00/0.00	95.65/0.67	94.97/0.97	94.97/0.97	–

*Red-color highlighted pairs show the highly conserved structural overlaps*.

#### Interaction partner analysis of GPXs

**Figure 4:**
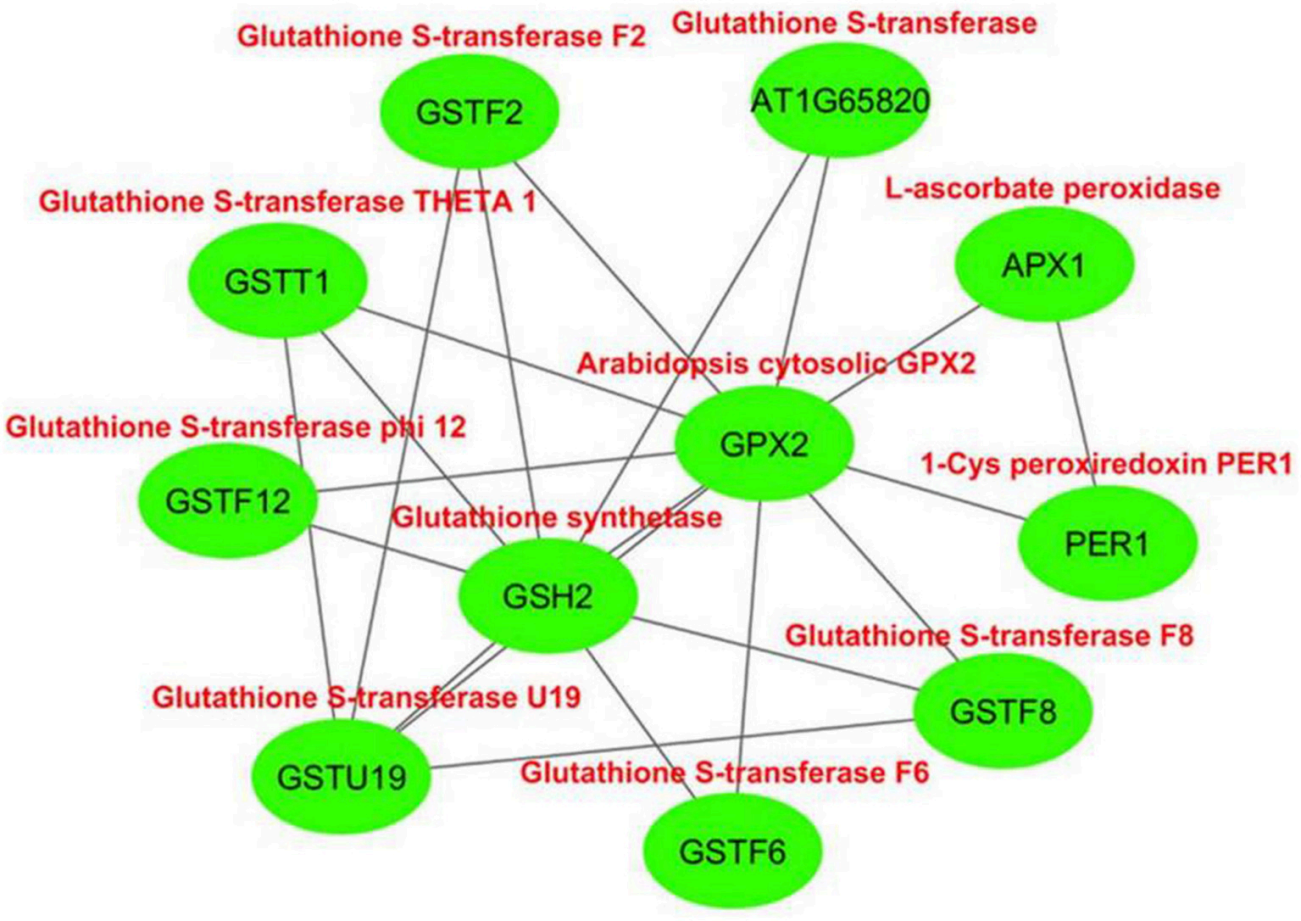
**Predicted 10 interaction partners of ***Arabidopsis*** cytosolic glutathione peroxidase GPX2**. Interactome was generated using cytoscape with STRING data. APX1 (L-ascorbate peroxidase), GSH2 (glutathione synthetase), GSTF6 (glutathione S-transferase F6), GSTT1 (glutathione S-transferase THETA 1), PER1 (1-Cys peroxiredoxin PER1), AT1G65820 (glutathione S-transferase), GSTF12 (glutathione S-transferase phi 12), GSTF2 (glutathione S-transferase F2), GSTF8 (glutathione S-transferase F8), and GSTU19 (glutathione S-transferase U19) proteins were predicted as main interaction partners of *Arabidopsis* cytosolic GPX2.

The interactome network was constructed for 10 putative interactors of *Arabidopsis* cytosolic GPX2, using Cytoscape with STRING data (Figure 4). APX1 (L-ascorbate peroxidase), GSH2 (glutathione synthetase), GSTF6 (glutathione S-transferase F6), GSTT1 (glutathione S-transferase THETA 1), PER1 (1-Cys peroxiredoxin PER1), AT1G65820 (glutathione S-transferase), GSTF12 (glutathione S-transferase phi 12), GSTF2 (glutathione S-transferase F2), GSTF8 (glutathione S-transferase F8), and GSTU19 (glutathione S-transferase U19) proteins were predicted as the main interaction partners of *Arabidopsis* cytosolic GPX2. APX1 is a type of H_2_O_2_-scavenging enzyme and a central component in the reactive oxygen gene network (Storozhenko et al., 1998; Fourcroy et al., 2004). GSH2 involves in the second step of the glutathione synthesis pathway from L-cysteine and L-glutamate (Wang and Oliver, 1996). GSTF6 functions in camalexin biosynthesis, is involved in the conjugation of reduced glutathione to various exogenous/endogenous hydrophobic electrophiles, and has a detoxification role for certain herbicides (Su et al., 2011). GSTT1, GSTF8, and GSTU19 are reported to have glutathione S-transferase or peroxidase activity. They further conjugate the reduced glutathione to various exogenous/endogenous hydrophobic electrophiles and play a detoxification role for certain herbicides (Wagner et al., 2002). PER1 is an antioxidant protein involved in the inhibition of germination under stress (Haslekås et al., 1998). AT1G65820 is a glutathione S-transferase. GSTF12 is involved in the transport of anthocyanins and proanthocyanidins into the vacuole (Kitamura et al., 2004). GSTF2 plays a role in binding and transport of small bioactive products and defense-related compounds under stress (Smith et al., 2003). The analysis indicated that cytosolic GPX2 enzyme is closely related with various pathways involving in antioxidant and secondary metabolite metabolisms, thereby supporting the functional role of GPXs in H_2_O_2_-scavenging and plant defense.

### Analysis of APXs

#### Retrieval of APX genes/proteins

Eight potential *Arabidopsis* APX protein sequences such as APX1-6, APXT, and APXS, obtained from the UniProtKB / Swiss-Prot database of NCBI, were used as queries in Phytozome database to retrieve the very close homologs of APX sequences in 18 plant species. In the selection of APX homologs from blastp hits, a very strict criterion (only the highest hit sequence) was applied to avoid redundant sequences and alternative splices of the same gene. A total of 120 APX sequences were identified from the protein datasets of 18 plant species. These were 8 genes for *A. thaliana*, 7 genes for *B. distachyon*, 8 genes for *B. rapa*, 4 genes for *C. reinhardtii*, 5 genes for *C. sativus*, 7 genes for *E. grandis*, 7 genes for *G. max*, 8 genes for *G. raimondii*, 7 genes for *M. truncatula*, 6 genes for *O. sativa*, 6 genes for *P. vulgaris*, 5 genes for *P. patens*, 7 genes for *P. trichocarpa*, 6 genes for *P. persica*, 7 genes for *S. lycopersicum*, 8 genes for *S. bicolor*, 6 genes for *V. vinifera*, and 8 genes for *Z. mays* (Table 5). Then, genomic, transcript, CDS, and protein sequences of 120 identified APX sequences were retrieved for further analyses.

**Table 5:** **List of H_**2**_O_**2**_-scavenging enzyme ascorbate peroxidase (APX) homologs from 18 plant species and their primary gene/protein features**.

**Species name**	**Phytozome gene ID**	**Gene/protein features of GPX sequences**							
		**Protein domain family^a^**	**Domain family description**	**Exon no**.	**Protein length**	**MW (KDa)**	***Theor. pI***	**Localization CELLO^b^**	**Localization WoLF PSORT^b^**
*Arabidopsis thaliana* (L.) Heynh.	AT1G07890	Peroxidase (PF00141)	Peroxidase	8	250	27.5	5.72	Cyto	Cyto
	AT1G77490	Peroxidase (PF00141)	Peroxidase	12	426	46.0	6.81	Chlo	Chlo
	AT3G09640	Peroxidase (PF00141)	Peroxidase	9	251	28.0	5.87	Cyto	Cyto
	AT4G08390	Peroxidase (PF00141)	Peroxidase	10	372	40.4	8.31	Chlo	Chlo
	AT4G09010	Peroxidase (PF00141)	Peroxidase	10	349	37.9	8.59	Chlo/Mito	Chlo
	AT4G32320	Peroxidase (PF00141)	Peroxidase	10	329	36.2	8.99	Chlo	Chlo
	AT4G35000	Peroxidase (PF00141)	Peroxidase	9	287	31.5	6.47	Cyto	Cyto
	AT4G35970	Peroxidase (PF00141)	Peroxidase	9	279	30.8	8.80	Cyto/Nucl	Cyto
*Brachypodium distachyon* (L.) P.Beauv.	Bradi1g16510	Peroxidase (PF00141)	Peroxidase	9	256	27.7	5.28	Cyto	Cyto
	Bradi1g65820	Peroxidase (PF00141)	Peroxidase	9	250	27.4	5.71	Cyto	Cyto
	Bradi3g40330	Peroxidase (PF00141)	Peroxidase	11	329	35.4	6.36	Chlo	Chlo
	Bradi3g42340	Peroxidase (PF00141)	Peroxidase	9	289	31.5	7.70	Cyto/Chlo	Cyto
	Bradi3g45700	Peroxidase (PF00141)	Peroxidase	12	439	47.3	5.61	Chlo	Chlo
	Bradi5g10490	Peroxidase (PF00141)	Peroxidase	11	345	37.4	8.77	Chlo/Mito	Chlo
	Bradi5g20670	Peroxidase (PF00141)	Peroxidase	10	333	36.1	8.71	Mito	Chlo
*Brasica rapa* L.	Brara.A00250	Peroxidase (PF00141)	Peroxidase	8	280	31.0	7.69	Cyto	Cyto
	Brara.A03521	Peroxidase (PF00141)	Peroxidase	9	251	28.1	6.41	Cyto	Cyto
	Brara.C02583	Peroxidase (PF00141)	Peroxidase	9	348	37.9	8.59	Chlo/Mito	Chlo
	Brara.G03518	Peroxidase (PF00141)	Peroxidase	10	439	47.5	7.70	Chlo	Chlo
	Brara.I02406	Peroxidase (PF00141)	Peroxidase	10	354	38.8	7.12	Chlo/Mito	Chlo
	Brara.I05334	Peroxidase (PF00141)	Peroxidase	7	250	27.5	5.61	Cyto	Cyto
	Brara.K00318	Peroxidase (PF00141)	Peroxidase	9	287	31.7	6.67	Cyto	Cyto
	Brara.K00699	Peroxidase (PF00141)	Peroxidase	10	327	36.1	8.72	Chlo	Chlo
*Chlamydomonas reinhardtii* P.A.Dang.	Cre02.g087700	Peroxidase (PF00141)	Peroxidase	10	327	35.6	8.67	Mito/Chlo	Chlo/Mito
	Cre05.g233900	Peroxidase (PF00141)	Peroxidase	8	347	36.4	9.23	Chlo	Chlo/Mito
	Cre06.g285150	Peroxidase (PF00141)	Peroxidase	7	337	35.1	8.95	Chlo/Mito	Chlo/Mito
	Cre09.g401886	Peroxidase (PF00141)	Peroxidase	10	372	39.4	8.63	Chlo	Chlo
*Cucumis sativus* L.	Cucsa.060660	Peroxidase (PF00141)	Peroxidase	11	413	44.8	7.09	Chlo	Chlo
	Cucsa.162470	Peroxidase (PF00141)	Peroxidase	8	249	27.3	7.74	Chlo/Cyto	Nucl
	Cucsa.213340	Peroxidase (PF00141)	Peroxidase	9	249	27.3	5.43	Cyto	Cyto
	Cucsa.311620	Peroxidase (PF00141)	Peroxidase	11	368	40.2	7.67	Chlo	Chlo
	Cucsa.370590	Peroxidase (PF00141)	Peroxidase	9	286	31.4	6.41	Cyto	Cyto
*Eucalyptus grandis* W. Hill ex Maiden	Eucgr.A01180	Peroxidase (PF00141)	Peroxidase	9	249	27.4	6.07	Cyto	Cyto
	Eucgr.B02456	Peroxidase (PF00141)	Peroxidase	9	249	27.2	5.29	Cyto	Cyto
	Eucgr.C01740	Peroxidase (PF00141)	Peroxidase	9	369	39.6	8.44	Chlo	Chlo
	Eucgr.F00373	Peroxidase (PF00141)	Peroxidase	11	356	38.3	6.50	Chlo	Chlo
	Eucgr.F04344	Peroxidase (PF00141)	Peroxidase	12	446	48.2	8.71	Chlo	Chlo
	Eucgr.F04344	Peroxidase (PF00141)	Peroxidase	11	397	42.8	8.60	Chlo	Chlo
	Eucgr.I01408	Peroxidase (PF00141)	Peroxidase	9	287	31.5	6.67	Cyto/Chlo	Cyto
*Glycine max* (L.) Merr.	Glyma.04G248300	Peroxidase (PF00141)	Peroxidase	11	386	41.9	7.06	Chlo	Chlo
	Glyma.06G068200	Peroxidase (PF00141)	Peroxidase	10	319	34.2	7.56	Chlo	Chlo
	Glyma.06G114400	Peroxidase (PF00141)	Peroxidase	12	432	47.0	7.13	Chlo	Chlo
	Glyma.11G078400	Peroxidase (PF00141)	Peroxidase	9	280	31.1	9.08	Cyto/Mito	Cyto
	Glyma.12G032300	Peroxidase (PF00141)	Peroxidase	9	287	31.7	6.27	Cyto	Cyto
	Glyma.12G073100	Peroxidase (PF00141)	Peroxidase	9	250	27.1	5.65	Cyto	Cyto
	Glyma.14G177200	Peroxidase (PF00141)	Peroxidase	10	347	37.9	6.76	Extr/Mito/Chlo	Chlo
*Gossypium raimondii* Ulbr.	Gorai.002G196800	Peroxidase (PF00141)	Peroxidase	9	288	31.7	5.64	Cyto	Cyto
	Gorai.005G254100	Peroxidase (PF00141)	Peroxidase	9	288	31.9	6.67	Cyto	Cyto
	Gorai.009G104500	Peroxidase (PF00141)	Peroxidase	9	250	27.5	5.73	Cyto	Cyto
	Gorai.009G246900	Peroxidase (PF00141)	Peroxidase	11	385	41.7	8.89	Chlo	Chlo
	Gorai.009G420500	Peroxidase (PF00141)	Peroxidase	9	251	27.8	6.01	Cyto	Cyto
	Gorai.010G038200	Peroxidase (PF00141)	Peroxidase	11	355	38.8	7.53	Chlo	Chlo
	Gorai.010G051400	Peroxidase (PF00141)	Peroxidase	12	422	46.0	6.77	Chlo	Chlo
	Gorai.010G115200	Peroxidase (PF00141)	Peroxidase	10	334	36.2	8.17	Chlo	Chlo
*Zea mays* L.	GRMZM2G004211	Peroxidase (PF00141)	Peroxidase	9	290	32.0	7.72	Cyto/Mito	Cyto
	GRMZM2G006791	Peroxidase (PF00141)	Peroxidase	12	451	48.9	5.60	Chlo	Chlo
	GRMZM2G047968	Peroxidase (PF00141)	Peroxidase	7	223	23.7	9.01	Chlo/Cyto	Mito/Chlo
	GRMZM2G054300	Peroxidase (PF00141)	Peroxidase	9	250	27.3	5.56	Cyto	Cyto
	GRMZM2G120517	Peroxidase (PF00141)	Peroxidase	11	339	37.0	8.86	Mito	Chlo
	GRMZM2G137839	Peroxidase (PF00141)	Peroxidase	9	250	27.3	5.64	Cyto	Cyto
	GRMZM2G156227	Peroxidase (PF00141)	Peroxidase	10	351	38.3	8.62	Mito	Chlo
	GRMZM2G460406	Peroxidase (PF00141)	Peroxidase	8	289	31.6	7.73	Cyto/Chlo	Cyto
*Vitis vinifera* L.	GSVIVG01008846001	Peroxidase (PF00141)	Peroxidase	11	372	40	7.10	Chlo	Chlo
	GSVIVG01009079001	Peroxidase (PF00141)	Peroxidase	10	344	37.4	6.65	Extr/Chlo	Chlo
	GSVIVG01024035001	Peroxidase (PF00141)	Peroxidase	9	289	31.7	7.72	Chlo/Cyto	Cyto
	GSVIVG01025104001	Peroxidase (PF00141)	Peroxidase	9	250	27.5	5.71	Cyto	Cyto
	GSVIVG01025551001	Peroxidase (PF00141)	Peroxidase	9	253	27.9	5.43	Cyto	Cyto
	GSVIVG01035858001	Peroxidase (PF00141)	Peroxidase	10	330	35.9	6.47	Chlo/Cyto	Chlo
*Oryza sativa* L.	LOC_Os02g34810	Peroxidase (PF00141)	Peroxidase	12	478	51.1	5.36	Chlo	Chlo
	LOC_Os04g35520	Peroxidase (PF00141)	Peroxidase	11	359	38.3	8.76	Chlo	Chlo
	LOC_Os04g51300	Peroxidase (PF00141)	Peroxidase	11	353	38.1	8.67	Mito/Chlo	Chlo
	LOC_Os07g49400	Peroxidase (PF00141)	Peroxidase	9	251	27.1	5.18	Cyto	Cyto
	LOC_Os08g41090	Peroxidase (PF00141)	Peroxidase	10	331	35.5	6.95	Chlo	Chlo
	LOC_Os08g43560	Peroxidase (PF00141)	Peroxidase	9	291	31.7	7.74	Chlo/Cyto/Mito	Cyto
*Medicago truncatula* Gaertn.	Medtr3g088160	Peroxidase (PF00141)	Peroxidase	11	436	47.4	9.02	Chlo	Chlo
	Medtr3g088160	Peroxidase (PF00141)	Peroxidase	10	387	42.0	8.73	Chlo	Chlo
	Medtr3g107060	Peroxidase (PF00141)	Peroxidase	10	320	34.7	8.08	Chlo	Mito/Chlo
	Medtr4g061140	Peroxidase (PF00141)	Peroxidase	9	250	27.1	5.52	Cyto	Cyto
	Medtr4g073410	Peroxidase (PF00141)	Peroxidase	9	287	31.7	6.26	Cyto	Chlo/Cyto
	Medtr5g022510	Peroxidase (PF00141)	Peroxidase	9	281	31.4	8.74	Cyto	Cyto
	Medtr5g064610	Peroxidase (PF00141)	Peroxidase	10	353	38.9	8.18	Mito/Nucl	Chlo
*Physcomitrella patens* (Hedw.) Bruch & Schimp.	Phpat.001G070500	Peroxidase (PF00141)	Peroxidase	11	358	38.4	7.56	Chlo	Chlo
	Phpat.001G104200	Peroxidase (PF00141)	Peroxidase	9	300	32.6	7.01	Chlo	Cyto
	Phpat.001G162800	Peroxidase (PF00141)	Peroxidase	2	440	48.2	8.11	Chlo	Chlo
	Phpat.017G025400	Peroxidase (PF00141)	Peroxidase	11	357	38.4	6.15	Chlo	Chlo
	Phpat.020G011100	Peroxidase (PF00141)	Peroxidase	9	250	27.6	5.66	Cyto	Cyto
*Phaseolus vulgaris* L.	Phvul.008G176700	Peroxidase (PF00141)	Peroxidase	10	347	37.6	6.05	Chlo/Extr	Chlo
	Phvul.009G093000	Peroxidase (PF00141)	Peroxidase	10	317	34.2	8.38	Chlo	Chlo
	Phvul.009G126500	Peroxidase (PF00141)	Peroxidase	12	436	47.8	8.67	Chlo	Chlo
	Phvul.009G126500	Peroxidase (PF00141)	Peroxidase	11	387	42.4	8.51	Chlo	Chlo
	Phvul.011G035000	Peroxidase (PF00141)	Peroxidase	9	287	31.6	7.10	Cyto	Cyto
	Phvul.011G071300	Peroxidase (PF00141)	Peroxidase	9	250	27	5.54	Cyto	Cyto
*Populus trichocarpa* Torr. & A.Gray ex. Hook.	Potri.004G174500	Peroxidase (PF00141)	Peroxidase	9	286	31.5	6.67	Cyto	Cyto
	Potri.005G161900	Peroxidase (PF00141)	Peroxidase	10	347	37.8	7.59	Chlo/Mito	Chlo
	Potri.005G179200	Peroxidase (PF00141)	Peroxidase	10	345	37.8	5.98	Cyto	Chlo/Mito
	Potri.006G132200	Peroxidase (PF00141)	Peroxidase	9	249	27.4	5.27	Cyto	Cyto
	Potri.006G254500	Peroxidase (PF00141)	Peroxidase	10	337	36.7	8.44	Chlo	Chlo
	Potri.009G015400	Peroxidase (PF00141)	Peroxidase	9	249	27.3	5.53	Cyto	Cyto
	Potri.009G134100	Peroxidase (PF00141)	Peroxidase	9	286	31.4	7.06	Cyto	Cyto
*Prunus persica (L.)* Batsch	ppa006270m	Peroxidase (PF00141)	Peroxidase	11	420	45.4	8.48	Chlo	Chlo
	ppa008008m	Peroxidase (PF00141)	Peroxidase	10	349	38.4	6.09	Mito/Chlo/Extr	Chlo
	ppa009582m	Peroxidase (PF00141)	Peroxidase	9	286	31.4	6.21	Cyto	Cyto
	ppa010413m	Peroxidase (PF00141)	Peroxidase	9	250	27.3	5.76	Cyto	Cyto
	ppa010426m	Peroxidase (PF00141)	Peroxidase	9	250	27.6	5.37	Cyto	Cyto
	ppa015878m	Peroxidase (PF00141)	Peroxidase	10	319	34.3	6.24	Chlo	Chlo
*Sorghum bicolor* (L.) Moench	Sobic.001G410200	Peroxidase (PF00141)	Peroxidase	9	250	27.2	5.55	Cyto	Cyto
	Sobic.002G431100	Peroxidase (PF00141)	Peroxidase	9	250	27.1	5.18	Cyto	Cyto
	Sobic.004G175500	Peroxidase (PF00141)	Peroxidase	13	473	51.1	5.03	Chlo	Chlo
	Sobic.006G021100	Peroxidase (PF00141)	Peroxidase	9	476	52.1	8.97	Nucl	Chlo
	Sobic.006G084400	Peroxidase (PF00141)	Peroxidase	11	344	37.2	8.60	Mito/Chlo	Chlo
	Sobic.006G204000	Peroxidase (PF00141)	Peroxidase	11	395	42.9	8.74	Mito/Chlo	Chlo
	Sobic.007G177000	Peroxidase (PF00141)	Peroxidase	8	289	31.5	7.73	Cyto	Cyto
	Sobic.007G205600	Peroxidase (PF00141)	Peroxidase	10	333	36.2	7.58	Chlo	Chlo
*Solanum lycopersicum* L.	Solyc01g111510	Peroxidase (PF00141)	Peroxidase	8	287	31.6	7.10	Cyto	Cyto
	Solyc04g074640	Peroxidase (PF00141)	Peroxidase	10	345	37.6	7.60	Chlo/Mito	Chlo
	Solyc06g005150	Peroxidase (PF00141)	Peroxidase	9	250	27.3	5.86	Cyto	Cyto
	Solyc06g060260	Peroxidase (PF00141)	Peroxidase	10	345	37.8	8.48	Chlo	Chlo
	Solyc08g059760	Peroxidase (PF00141)	Peroxidase	10	326	35.4	5.65	Chlo	Chlo
	Solyc09g007270	Peroxidase (PF00141)	Peroxidase	9	250	27.6	5.63	Cyto	Cyto
	Solyc11g018550	Peroxidase (PF00141)	Peroxidase	12	421	46.0	8.20	Chlo	Chlo

a *Protein domain families were searched in Pfam database*.

b *Cyto, Cytosolic; Chlo, Chloroplastic; Mito, Mitochondrial; Nucl, Nuclear; Extr, Extracellular*.

*More than one localization specified in a single column also shows the significance of other entries in order*.

#### Sequence analysis of APX genes/proteins

A total of 120 APX homologs were identified in protein datasets of 18 plant species using *Arabidopsis* APX1-6, APXT, and APXS sequences by homology search. Identified APX sequences contained the peroxidase (PF00141) protein family domain. They encoded a protein of 197–478 amino acids residues (average length 323.9) and 23.7–52.1 kDa molecular weight with 5.03–9.23 *pI* value. The sequence variations in analyzed APXs demonstrated a correlation with their putative localizations, thereby indicated the presence of transit residues (Table 5). Molecular cloning studies from *A. thaliana* have demonstrated that APX1, APX2, and APX6 are polypeptides of 250, 251, and 329 amino acids, respectively, with cytosolic localizations but without transit peptide (Davletova et al., 2005; Jones et al., 2009; Aryal et al., 2011). APX3 and APX5 consisted of 287 and 279 amino acids, respectively, with peroxisomal localizations; however, sites of transit peptide residues are not precisely specified (Panchuk et al., 2002; Narendra et al., 2006; Bienvenut et al., 2012). APX4 is a 349 amino acids protein with chloroplastic localization, including 1–82 residues as transit peptide from the N-terminal site (Kieselbach et al., 2000; Panchuk et al., 2005; Aryal et al., 2011). APXT is a 426 amino acids chloroplastic protein, including 1–78 residues of transit peptide (Theologis et al., 2000; Panchuk et al., 2005). APXS consists of 372 amino acids with chloroplastic and/or mitochondrial localizations, but the exact site of the transit peptide is not specified (Jespersen et al., 1997; Mayer et al., 1999; Chew et al., 2003). In the present study, multiple-alignment of APX sequences revealed that chloroplastic/mitochondrial-related APXs contained the transit peptide residues in approximately 1–90 amino acids from the N-terminal site while cytosolic APXs did not have any putative transit peptide (Supplementary Figure S4). Thus, the analyzed APX sequences were gathered in two main groups based on subcellular localizations, such as chloroplastic/mitochondrial APXs (i) and cytosolic APXs (ii).

In addition, the regions corresponding to the transit peptide sites in analyzed sequences did not demonstrate any particular pattern. This could indicate that less conservancy in transit peptides may be associated with the functional diversities of APXs at various targets. Besides, APX transcripts mainly consisted of 8–12 exons, supporting the relatively less conserved structure of APXs compared to GPXs. However, alignment analysis also demonstrated the presence of a considerable degree of conserved residues in the main sites of enzyme (Supplementary Figure S5). Moreover, to analyze the availability of any conserved motif pattern/s in APX sequences, the most conserved five motif sequences of APX homologs were searched using MEME tool (Table 6). Motif 1 was 29 residues long, motif 2 and 4 were 21 residues, motif 3 was 32 residues, and motif 5 was 25 residues in length. However, only motifs 2 and 3 had a relation with the peroxidase (PF00141) protein family, and in this case were present in most of the sequences. This could indicate the highly conserved structures of APX sequences at those sites with peroxidase activity.

**Table 6:** **Most conserved five motifs of ascorbate peroxidase (APX) homologs in 18 plant species**.

**Motif**	**Width**	**Identified site no**.	**Sequence**	**Protein domain family^a^**
1	29	120 of 120	CHPIMLRLAWHDAGTYDKNTKTWGPNGSI	Not found
2	21	101 of 120	MGLNDQDIVALSGGHTLGRCH	Peroxidase (PF00141)
3	32	119 of 120	IITYADLYQLAGVVAVEVCGGPTIPMHCGRND	Peroxidase (PF00141)
4	21	118 of 120	DPEFRPWVEKYAEDQDAFFRD	Not found
5	25	84 of 120	ERSGFEQPWTVNWLKFDNSYFKEIL	Not found

a *Protein domain families have been searched in Pfam database*.

Furthermore, alignment analysis also demonstrated that Asp (D) and Gly-Gly (GG) residues are strictly conserved in all aligned sequences, indicating their potential functions in enzyme activity and/or stability (Supplementary Figure S6). To infer a functional relationship between these conserved residues and APX sequences, we searched for the known binding residues of model organism *Arabidopsis* APXs in the UniProtKB database (http://www.uniprot.org/uniprot/). The following residues were reported as potential active and metal binding residues for *Arabidopsis* GPX1-6, APXT, and APXS: APX1 (Arg-38, His-42, His-163, Thr-164, Thr-180, Asn-182, Ile-185, Asp-187), APX2 (Arg-39, His-43, His-163, Thr-164, Thr-180, Asn-182, Ile-185, Asp-187), APX3 (Arg-36, His-40, His-160, Thr-161, Thr-177, Asp-184), APX5 (Arg-35, His-39, His-158, Thr-159, Thr-175, Asp-182), APX6 (Arg-119, His-123, His-224), APXT (Arg-108, His-112, His-241, Thr-242, Thr-274, Asp-281), and APXS (Arg-129, His-133, His-262, Thr-263, Thr-295, Asp-302). These active and metal binding residues did not correspond to any of the strictly conserved residues in analyzed APX sequences but they were found to be conserved at considerable rates. However, when taken into consideration that some of the strictly conserved residues in analyzed GPX sequences correspond to the catalytic sites of the enzymes, we can make an inference that these strictly conserved residues in APX sequences may also be associated with the peroxidase activity of the enzyme.

#### Phylogenetic analysis of APXs

**Figure 5:**
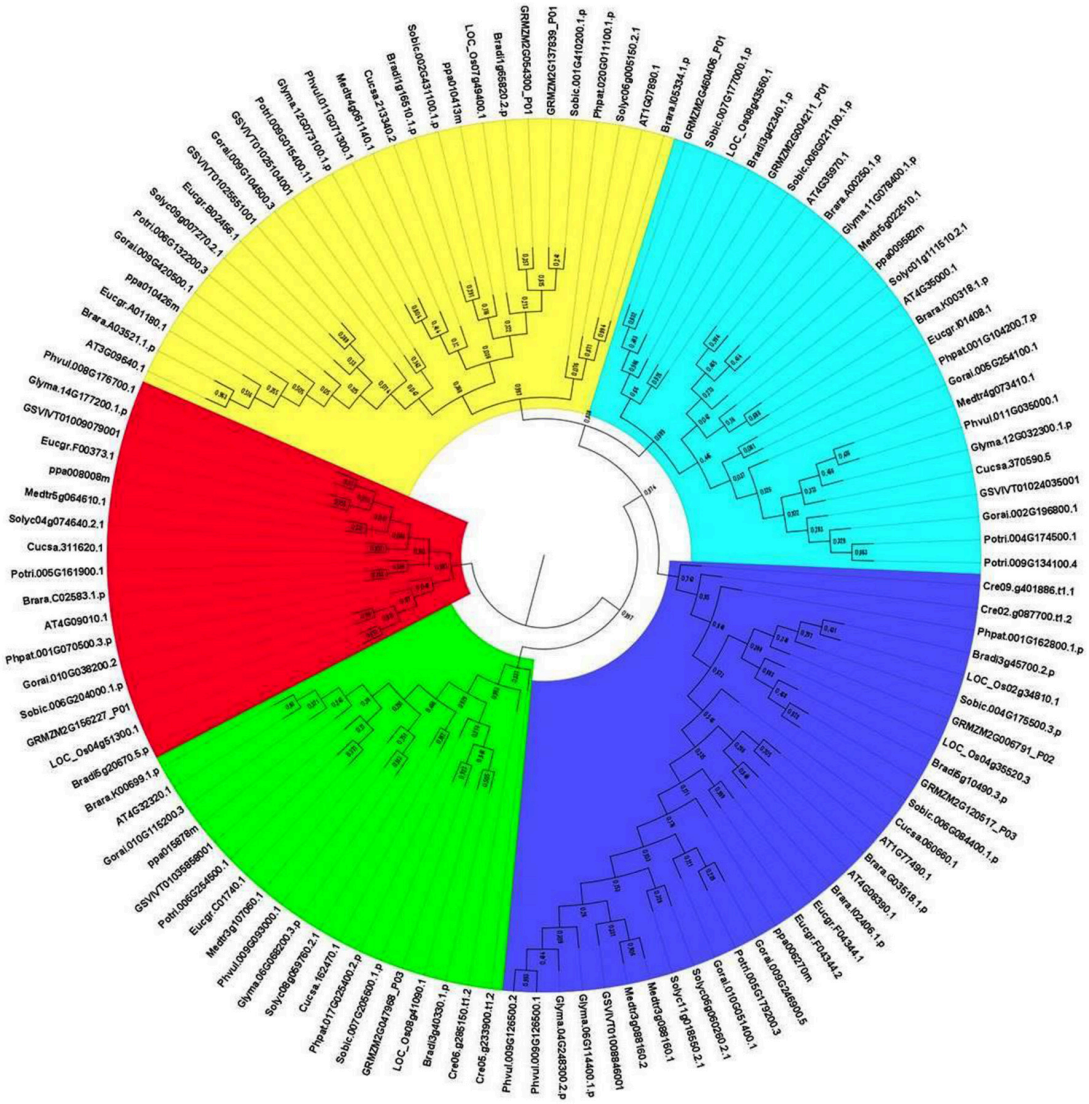
**Phylogenetic tree of ascorbate peroxidase (APX) homologs from 18 plant species**. Tree was constructed by MEGA 6 using Maximum likelihood (ML) method with 1000 bootstraps. Segment classification based on the consensus of two subcellular localization servers, CELLO and WoLF PSORT as well as tree topology for ambiguous sequences. Blue, red, and green segments include chloroplast/mitochondrial related APXs with mainly longer, medium and short sequences, respectively, while cyan and yellow segments contain longer and shorter cytosolic APX sequences, respectively.

To analyze the evolutionary relationship between identified APX homologs, the phylogenetic tree was constructed by MEGA 6 using the Maximum Likelihood (ML) method with 1000 bootstraps (Figure 5). The constructed tree was divided into five major groups based on the tree topology, and each group was indicated with a different color segment. Blue, red, and green segments included the chloroplast/mitochondria-related APXs with relatively longer, medium and short sequences, respectively, whereas cyan and yellow segments mainly contained longer and shorter cytosolic APX sequences, respectively. Annotation of each segment was based on the consensus of two subcellular localization servers, CELLO and WoLF PSORT, as well as tree topology for ambiguous sequences. Overall, it was observed that cytosolic-related APXs clustered together, while alternatively chloroplast/mitochondrial-related APXs were together. In addition, in clustering of sequences at sub-branches was primarily based on the sequence length and monocot/dicot separation. However, there were considerable variations between sequences, even those belonging to the same subcellular localization. It is thought that these sequence variations could be attributed to the various functional diversities of APXs and/or be associated with different subcellular localizations. Moreover, some sequences were also available with different subcellular localizations in the same clade, indicating the possibility of gene duplication events in formation of some *APX* genes. The gene duplication events were searched based on the previously designated protocol (Gu et al., 2002). In doing so, several segmental and tandem duplications were identified between some APX pairs (Table 7). The identified segmental or tandem duplications in *APX* genes were observed between either chloroplastic and chloroplastic, or cytosolic and cytosolic forms. This could indicate the possibility of gene duplication events in the formation of close *APX* homologs.

**Table 7:** **The segmental and tandem duplications in some ascorbate peroxidase (APX) pairs**.

**Duplication type**	**Species name**	**Duplicated pairs**
Segmental duplication Pairs	*Brachypodium distachyon* (L.) P.Beauv.	Bradi5g10490-Bradi3g45700
	*Eucalyptus grandis* W. Hill ex Maiden	Eucgr.A01180-Eucgr.B02456
	*Glycine max* (L.) Merr.	Glyma.06G114400-Glyma.04G248300
		Glyma.11G078400-Glyma.12G032300
	*Gossypium raimondii* Ulbr.	Gorai.002G196800-Gorai.005G254100
		Gorai.009G246900-Gorai.010G051400
	*Vitis vinifera* L.	GSVIVG01025104001-
		GSVIVG01025551001
	*Oryza sativa* L.	LOC_Os04g35520-LOC_Os02g34810
	*Populus trichocarpa* Torr. & A.Gray ex. Hook.	Potri.004G174500-Potri.009G134100
		Potri.006G132200-Potri.009G015400
	*Prunus persica* (L.) Batsch	ppa010431m.g-ppa010426m.g
	*Sorghum bicolor* (L.) Moench	Sobic.001G410200-Sobic.002G431100
		Sobic.006G084400-Sobic.004G175500
		Sobic.007G177000-Sobic.006G021100,
	*Solanum lycopersicum* L.	Solyc06g005150.2-Solyc09g007270.2
		Solyc06g060260.2-Solyc11g018550.2
Tandem duplication Pairs	*Brachypodium distachyon (L.) P.Beauv*.	Bradi1g16510-Bradi1g65820
	*Gossypium raimondii Ulbr*.	Gorai.009G104500-Gorai.009G420500
	*Zea mays L*.	GRMZM2G006791-GRMZM2G120517
		GRMZM2G054300-GRMZM2G137839

#### Expression profile analysis of APXs

**Figure 6:**
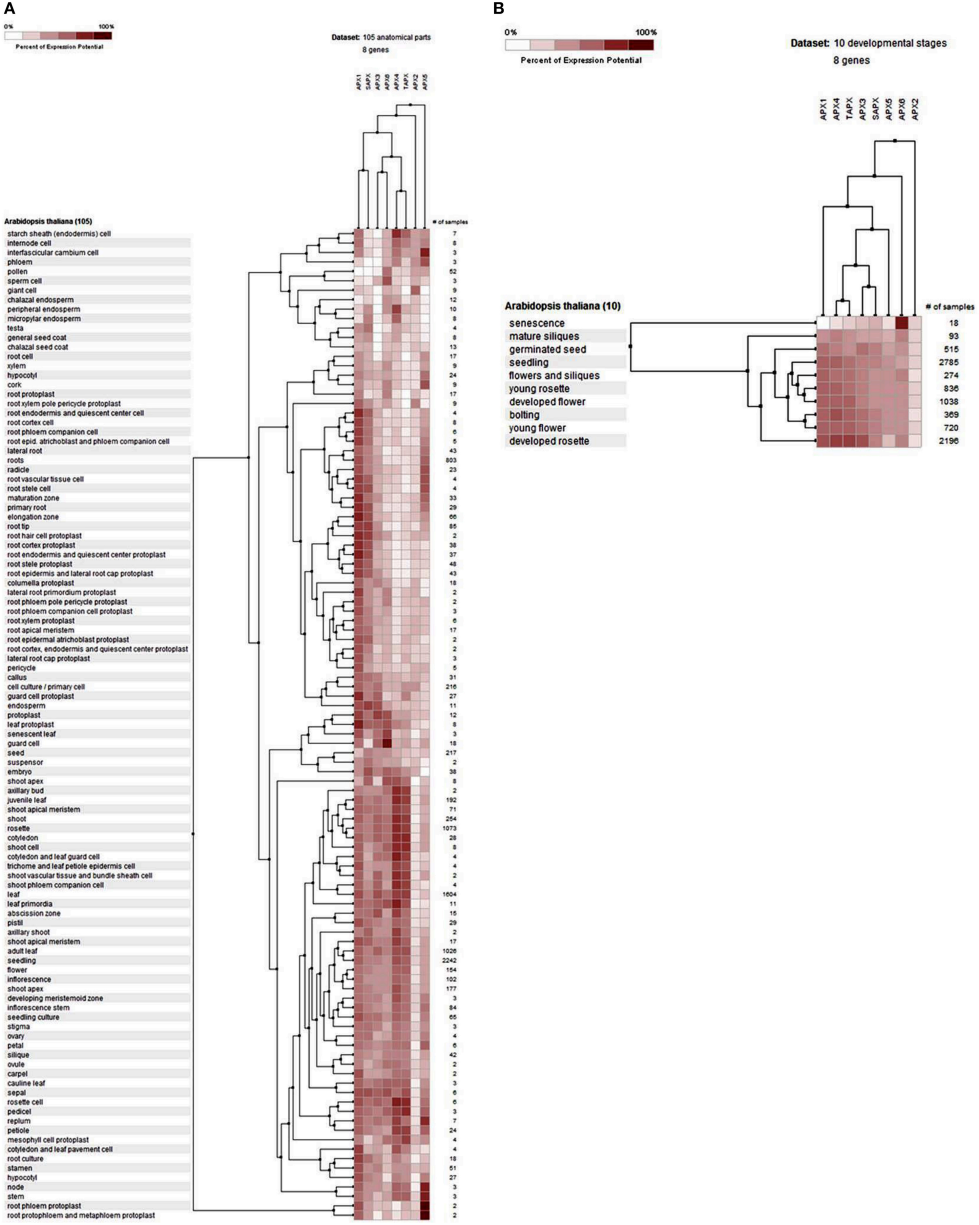
**Expression profile of ***Arabidopsis*** ascorbate peroxidase ***APX1-6, TAPX*** and ***SAPX*** genes at 105 anatomical parts (A) and 10 developmental stage levels (B)**. Genes and conditions with similar profiles were comparatively analyzed using hierarchical clustering tool with Euclidean distance method at Genevestigator platform.

The gene expression profiles of *APX*s were analyzed at 105 anatomical parts and 10 developmental stage levels using model organism *A. thaliana APX*s from Genevestigator platform (Figure 6). Eight *Arabidopsis* genes, namely APX1 (AT1G07890), APX2 (AT3G09640), APX3 (AT4G35000), APX4 (AT4G09010), APX5 (AT4G35970), APX6 (AT4G32320), TAPX (AT1G77490), and SAPX (AT4G08390), were retrieved from the “Affymetrix Arabidopsis ATH1 Genome Array” platform using the Genevestigator interface. Thereafter, conditions and genes with similar profiles were comparatively analyzed using Hierarchical clustering tool with Euclidean distance method.

At the anatomical level (Figure 6A), *APX* genes were expressed in almost all analyzed tissues of *Arabidopsis* with various folds. It was clear that the expression levels of genes were closely related with the expressed tissue type/s. For example, both cytosolic *APX1* and chloroplastic/mitochondrial *SAPX* had significantly higher expression in actively growing zones, as well as many root and root protoplast-related structures. *APX3, APX4, APX6*, and *TAPX* were expressed in various shoot, bud, leaf, flower and seed related tissues at considerable rates. All these indicated that stress factors, actively growing tissues as well as normal physiological and metabolic changes could induce the expression of *APX* genes in tissue-dependent way. All these metabolic activities or their related consequences could exert the stresses to the cells. Many studies have further demonstrated that abiotic/abiotic stress factors such as heavy metal, drought, water, heat, cellular H_2_O_2_ level, oxidative state of the cell could increase the expression of *APX* genes to either suppress or eliminate the stressors (Ishikawa and Shigeoka, 2008; Koussevitzky et al., 2008; Yang et al., 2008; Petrov and Van Breusegem, 2012). For example, overexpression of *Solanum melongena APX6* in transgenic *O. sativa* seedlings demonstrated high flood tolerance, reduced oxidative injury and fast plant growth rates (Chiang et al., 2015a). APX regulation by nitric oxide (NO) as a redox indicator in oxidative stress or as part of hormone induced signaling pathway in lateral root development were demonstrated (Correa-Aragunde et al., 2015). *S*-nitrosylation of *Arabidopsis APX1* at Cys32 increased the H_2_O_2_ scavenging activity of enzyme, resulting in improved oxidative stress tolerance (Yang et al., 2015). Overexpression of *APX* and *Cu/Zn SOD* increased the drought resistance and recovery capacity from drought stress in *Ipomoea batatas* (Lu et al., 2015). Overexpressed *Brassica campestris* APX gene in transgenic *Arabidopsis* enhanced the heat tolerance *via* elimination of H_2_O_2_ (Chiang et al., 2015b). Therefore, increased APX activity in cells is an indicator of the presence of stress factors. At the developmental level (Figure 6B), the expression profile of *Arabidopsis APXs* was analyzed at 10 developmental stages: senescence, mature siliques, flowers and siliques, developed flower, young rosette, germinated seed, seedling, bolting, young flower, and developed rosette. In all developmental stages, *APXs* were relatively expressed. However, the expression pattern in senescence was slightly different from other developmental stages, notably cytosolic *APX6* showed the highest expression. Interestingly, the *Arabidopsis GPX6* gene also demonstrated the highest expression fold at senescence stage, inferring the possibility of functional similarities of these two enzymes. Overall, the expression profile and fold of *APX*s in various tissues and stages show that cells are constantly put under stress even with normal physiological and metabolic changes, requiring plants to eliminate these stressors.

#### 3D structure analysis of APXs

**Figure 7:**
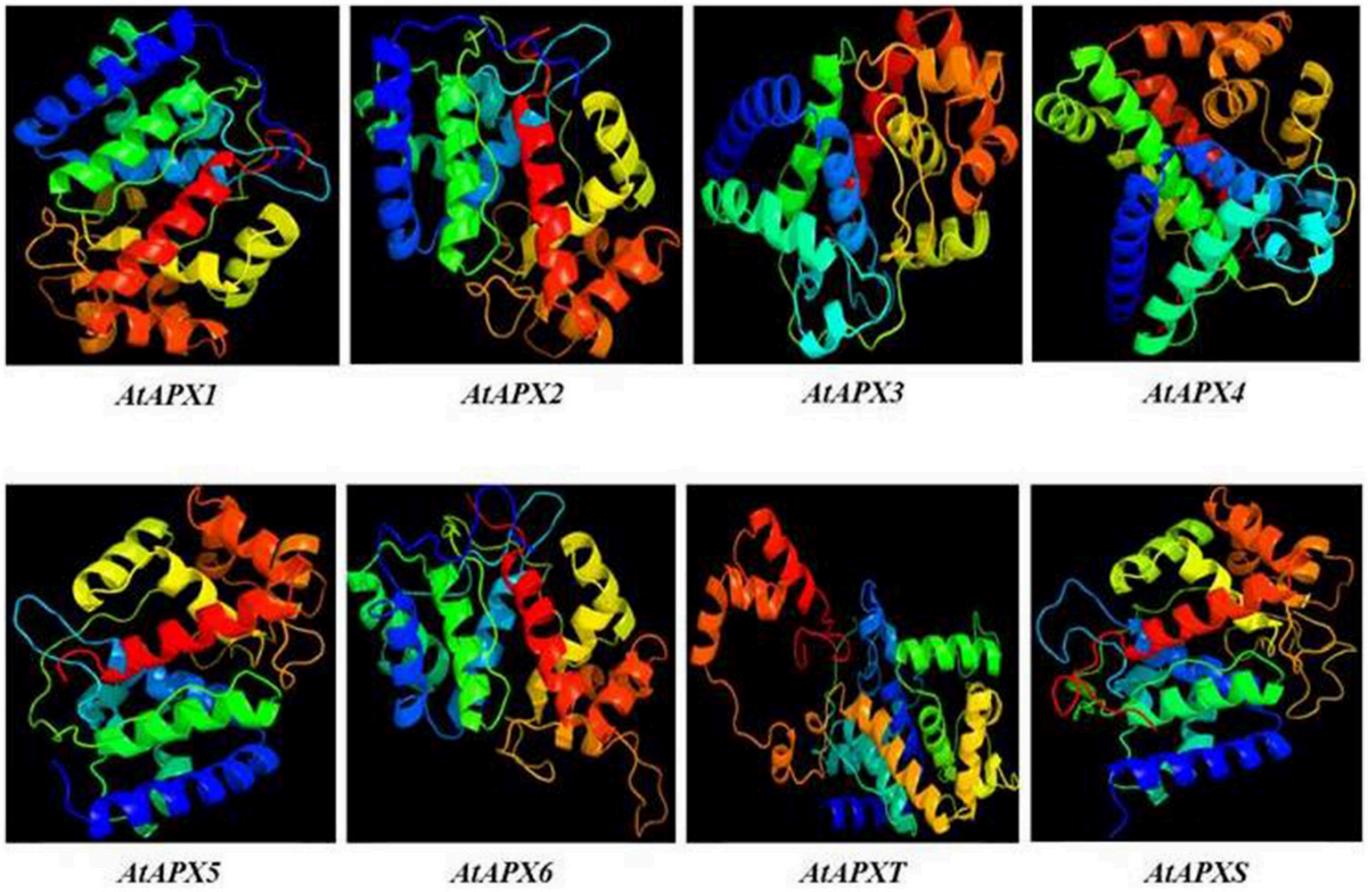
**3D models of predicted ***Arabidopsis*** ascorbate peroxidase APX1-6, APXT, and APXS sequences**. Models were constructed by using Phyre^2^ server for AT1G07890.1 (APX1), AT3G09640.1 (APX2), AT4G35000.1 (APX3), AT4G09010.1 (APX4), AT4G35970.1 (APX5), AT4G32320.1 (APX6), AT1G77490.1 (APXT), and AT4G08390.1 (APXS) sequences, and colored by rainbow from N- to C-terminus.

3D models of eight identified *Arabidopsis* APX sequences were constructed by using Phyre2 server (Figure 7). The modeled sequences were AT1G07890.1 (*APX1*), AT3G09640.1 (*APX2*), AT4G35000.1 (*APX3*), AT4G09010.1 (*APX4*), AT4G35970.1 (*APX5*), AT4G32320.1 (*APX6*), AT1G77490.1 (*APXT*), and AT4G08390.1 (*APXS*). In modeling, six templates such as 1APX:A (*APX1*), 1OAF:A (*APX2, APX3* and *APX5*), 3RRW:B (*APX4*), 1BGP:A (*APX6*), 1ITK:B (*APXT*), and 1IYN:A (*APXS*) were used to maximize the alignment coverage, percentage identity, and confidence for the submitted sequences. Predicted models showed the ≥96% of residues were within the allowed region in Ramachandran plot, indicating that structures were acceptably high in quality. To analyze the divergence or similarity in generated models, the structures were superposed in order to calculate the percentage of structural overlap and RMSD values (Table 8). The superposition of APX sequences demonstrated that APX2-APX3, APX2-APX5, and APX3-APX5 pairs have highly conserved structural overlap (100%) with 0.00, 0.38, and 0.38 RMSD values, respectively. These conserved pairs primarily shared the cytosolic and/or peroxisomal localizations, inferring the possibility of a functional relationship between them. In addition, the APX1-APX2, 3, and 5 pairs had very high structural similarity with ≥99 structural overlaps. Therefore, it could be deduced that APX members topologically demonstrated highly conserved structures, despite their functional diversities in different cellular compartments.

**Table 8:** **Structural overlap (%)/RMSD values in superposed ***Arabidopsis*** ascorbate peroxidases (APXs)**.

	**APX1**	**APX2**	**APX3**	**APX4**	**APX5**	**APX6**	**APXS**	**APXT**
**APX1**	–	99.19/0.43	99.59/0.41	75.10/1.75	99.58/0.51	81.53/1.52	95.18/0.95	89.16/1.49
**APX2**	99.19/0.41	–	100.00/0.00	75.0071.78	100.00/0.38	81.45/1.58	95.16/0.86	87.90/1.35
**APX3**	99.59/0.41	100.00/0.00	–	75.31/1.85	100.00/0.38	82.30/1.56	97.12/0.86	88.48/1.38
**APX4**	75.10/1.75	75.40/1.73	73.66/1.91	–	73.22/1.92	72.29/1.88	75.79/1.72	66.27/1.83
**APX5**	99.58/0.48	100.00/0.38	100.00/0.38	74.48/1.85	–	81.17/1.67	97.49/0.90	89.12/1.31
**APX6**	82.73/1.57	82.26/1.70	83.13/1.68	69.88/1.82	84.10/1.70	–	81.12/1.50	73.90/1.66
**APXS**	95.18/1.00	95.97/0.97	97.12/1.00	75.00/1.73	97.49/1.05	82.73/1.55	–	83.52/1.38
**APXT**	87.55/1.47	89.52/1.36	89.71/1.32	67.46/1.87	90.79/1.32	74.70/1.80	82.42/1.34	–

*Red-color highlighted pairs show the highly conserved structural overlaps*.

#### Interaction partner analysis of APXs

**Figure 8:**
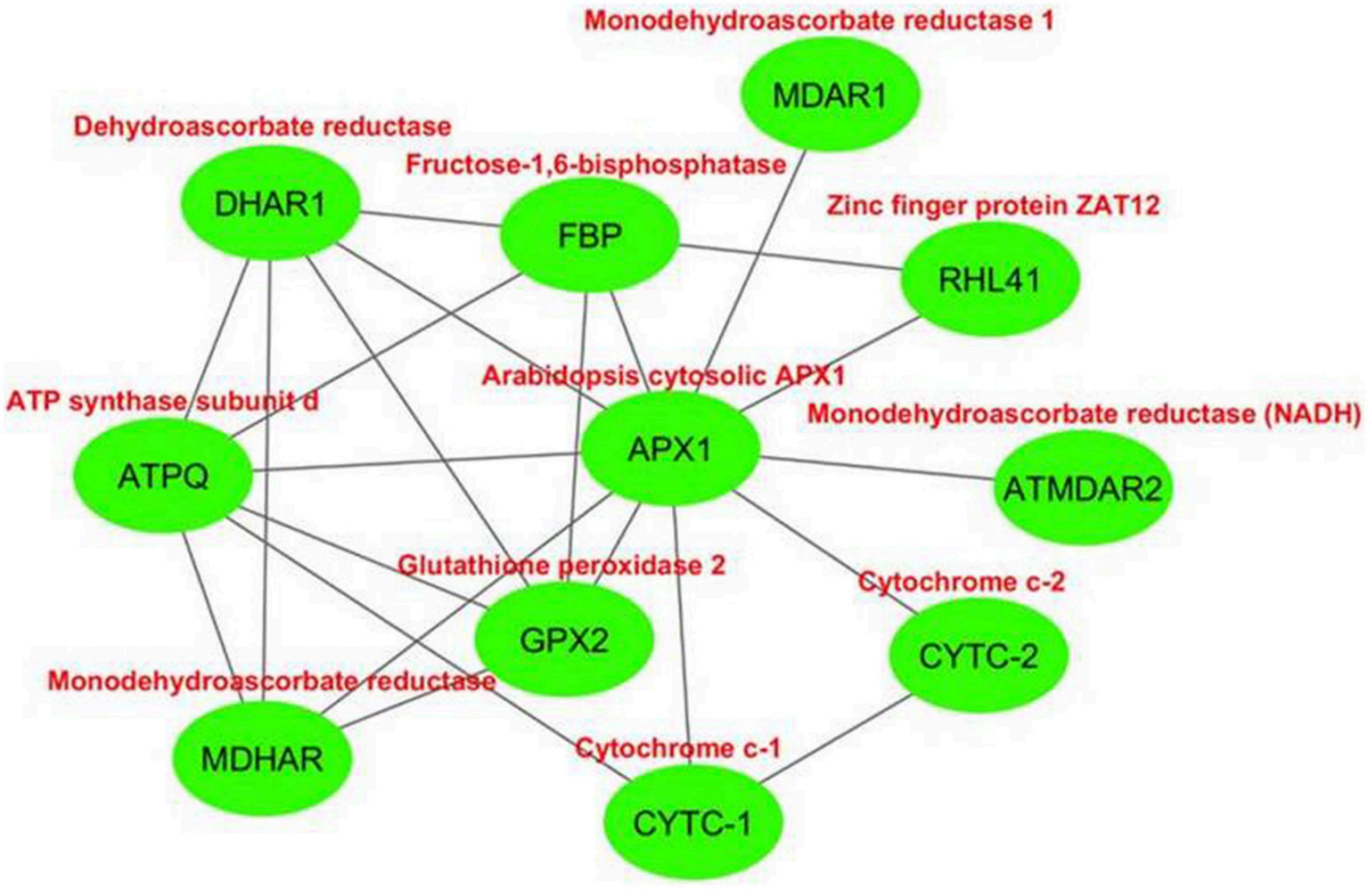
**Predicted 10 interaction partners of ***Arabidopsis*** cytosolic ascorbate peroxidase APX1**. Interactome was generated using cytoscape with STRING data. MDHAR (monodehydroascorbate reductase), GPX2 (glutathione peroxidase 2), DHAR1 (dehydroascorbate reductase), MDAR1 (monodehydroascorbate reductase 1), RHL41 (zinc finger protein ZAT12), ATPQ (ATP synthase subunit d), FBP (fructose-1,6-bisphosphatase), ATMDAR2 (monodehydroascorbate reductase (NADH)), CYTC-1 (cytochrome c-1), and CYTC-2 (cytochrome c-2) proteins were predicted as main interaction partners of *Arabidopsis* cytosolic APX1.

The interactome network was constructed for 10 putative interactors of *Arabidopsis* cytosolic APX1 using Cytoscape with STRING data (Figure 8). MDHAR (monodehydroascorbate reductase), GPX2 (glutathione peroxidase 2), DHAR1 (dehydroascorbate reductase), MDAR1 (monodehydroascorbate reductase 1), RHL41 (zinc finger protein ZAT12), ATPQ (ATP synthase subunit d), FBP (fructose-1,6-bisphosphatase), ATMDAR2 [monodehydroascorbate reductase (NADH)], CYTC-1 (cytochrome c-1), and CYTC-2 (cytochrome c-2) proteins were predicted as the main interaction partners of *Arabidopsis* cytosolic APX1. MDHAR, MDAR1 and ATMDAR2 catalyze the conversion of monodehydroascorbate to ascorbate (Chew et al., 2003). GPX2 is a type of H_2_O_2_-scavenging enzyme and a crucial component in reactive oxygen network (Tanaka et al., 2005). DHAR1 has dual functions: soluble protein, it demonstrates GSH-dependent thiol transferase and dehydroascorbate (DHA) reductase activities, and is involved in redox homeostasis. As a peripheral membrane protein, it functions as voltage-gated ion channel (Dixon et al., 2002; Sasaki-Sekimoto et al., 2005). RHL41 affects in modulation of light acclimation, and cold and oxidative stress responses (Rizhsky et al., 2004; Davletova et al., 2005). ATPQ functions in ATP production (Carraro et al., 2014). FBP is reported to be a key component in photosynthetic sucrose synthesis (Cho et al., 2012). CYTC-1 and CYTC-2 are electron carrier proteins related with mitochondrial electron transport chain (Welchen and Gonzalez, 2005). In light of putative interaction partner analysis, it was apparent that *Arabidopsis* cytosolic APX1 is either directly or indirectly associated with redox homeostasis, stress adaptation and photosynthesis/respiration-related pathways. This could also help in better understanding the functional role of APX1 in various plant defense mechanisms.

### Comparison of APX and GPX sequences

A strict homology search of *Arabidopsis* GPX1-8 sequences in proteome datasets of 18 specified plant species has given a total of 87 putative GPX sequences; however, homology search of *Arabidopsis* APX1-6, APXT, and APXS in proteome datasets of these species identified a total of 120 putative APXs (Tables 1, 5). Sequences of GPX homologs contained the GPX (PF00255) protein family domain while APX homologs included the peroxidase (PF00141) domain. *GPX* genes encoded a protein of 166–262 residues with 18.4–29.7 kDa molecular weight and 4.59–9.60 *pI* value, while *APX*s encoded a polypeptide of 197–478 residues with 23.7–52.1 kDa molecular weight and 5.03–9.23 *pI* value. GPX transcripts mainly contained six exons; whereas, APX usually had 8–12 exons, implicating the relatively less conserved structure of APXs compared to GPXs. Sequence variations in GPX and APX homologs primarily derived from the “transit peptide” residues between organelle and non-organelle related sequences. Besides, regions corresponding to transit peptide sites in APX/GPX sequences did not demonstrate any particular pattern, indicating the less conserved structure of transit peptides thereby the functional diversities of APXs/GPXs at various targets. In addition, multiple-alignment analyses demonstrated the presence of a considerable degree of conserved residues in main sites of both enzymes. In GPX phylogeny, cytosolic-, nuclear-, extra cellular,- and plasma membrane-related GPXs were relatedly clustered while chloroplast/mitochondrial-related GPXs grouped together. APX phylogeny also showed similar clustering pattern, in which cytosolic-related APXs were relatedly clustered while chloroplast/mitochondrial-related APXs were together. This indicates that presence/absence of “transit peptide” residues was the main determinant in phylogenetic distribution of APX/GPX sequences. Moreover, presence of sequences with different subcellular localizations in the same phylogenetic group inferred the possibility of gene duplication events in formation of some APX/GPX sequences. Several segmental duplications were identified in some GPX pairs, while several segmental and tandem duplications were available in some APX pairs. Expression profiles of *GPX* and *APX* genes in model organism *Arabidopsis* indicated that stress factors, actively growing tissues, even normal physiological, and metabolic changes could induce the expression of *APX/GPX* genes. Interactome analyses of *Arabidopsis* cytosolic APX1 and GPX2 also implicated that both enzymes are closely related with antioxidant and redox homeostasis, secondary metabolite metabolisms and stress adaptation thereby supporting the functional roles of APXs/GPXs in H_2_O_2_-scavenging and plant defense. Despite of some minor variations, APX and GPX members, they topologically demonstrated highly conserved structure.

## Conclusions

The presence or absence of transit peptide residues are the main contributing factors in subcellular localization and phylogenetic distribution of APX/GPXs. The APX/GPX expression is highly associated with the metabolic state of the cells. In addition, there are grounds for belief that these two enzymes work together in various pathways such as antioxidant and secondary metabolite metabolisms, redox homeostasis, stress adaptation, and photosynthesis/respiration. This also supports the functional role of these enzymes in H_2_O_2_-scavenging, thereby implicating their importance in the plant defense. However, further molecular and physiological studies are required to elucidate the various functional roles of APX/GPX isoforms.

## Author contributions

IK and EF contributed to the study conception and design. KK performed experiments and collected data. Data analysis and interpretation were performed by RV. IK, EF, KK, and RV prepared, and NA performed critical reading and revision of the manuscript. IO and EF supervised and MO coordinated this work. All the authors read and approved the final version.

### Conflict of interest statement

The authors declare that the research was conducted in the absence of any commercial or financial relationships that could be construed as a potential conflict of interest.
