# Supplementary material for: Identification and Comparative Analysis of H2O2-Scavenging Enzymes (Ascorbate Peroxidase and Glutathione Peroxidase) in Selected Plants Employing Bioinformatics Approaches
Source: Front Plant Sci. 2016 Mar 22;7:301. doi: 10.3389/fpls.2016.00301 (PMC4802093; doi:10.3389/fpls.2016.00301)
Supplement: Supplementary file 6 [file DataSheet6.DOC]

**Supplementary Figure S6 (Above).** Multiple alignment of 120 APX homologous from 18 plant species. Identical and similar resides were shaded as black and grey, respectively with 100% threshold value to find out the strictly conserved residues between intra- and interspecies.
